# Utility of HEARTSMAP-U for psychosocial screening and mental health resource navigation in the young adult population

**DOI:** 10.1371/journal.pone.0353390

**Published:** 2026-07-13

**Authors:** Shane Murphy, Delnaz Dadkhah Tirani, Jillian Wagg, Karly Stillwell, Liz Lee, Quynh Doan

**Affiliations:** 1 BC Children’s Hospital Research Institute, Vancouver, British Columbia, Canada; 2 Department of Pediatrics, University of British Columbia, Vancouver, British Columbia, Canada; NYU Grossman School of Medicine: New York University School of Medicine, UNITED STATES OF AMERICA

## Abstract

**Purpose:**

Given the high prevalence of mental health struggles and long wait times for psychological assessment, validated digital self-assessment screening tools can help to address the gap. Our previously validated tool for ages 6–17, MyHEARTSMAP, was adapted to focus on the transition to university. This new tool, HEARTSMAP-U, captures the issues of older youth, addresses student-specific psychosocial needs, and recommends resources matching the type and urgency of their reported needs. Prior to widespread use in the public domain, we aim to validate the psychometric properties of HEARTSMAP-U as compared to a standard mental health intake assessment.

**Methods:**

We conducted a prospective validation study of students at a Canadian university aged 17–29. Participants completed psychosocial self-assessments using HEARTSMAP-U, which were then directly compared to equivalent, standardized assessments conducted by clinical counselors. We reported the sensitivity and specificity of the participant self-assessments against that of a clinician’s intake evaluation. This considered each respondent’s degree of unmet needs, and through this, each person was provided with a catered list of local supports for each psychosocial domain.

**Result:**

Of the 619 eligible participants, 536 completed baseline evaluations for analysis. Using HEARTSMAP-U, post-secondary students’ sensitivity of self-identifying any degree of psychiatric concern was 90% (95% CI 83–94%). When clinicians identified no psychiatric concerns, HEARTSMAP-U self-assessments similarly identified either no or mild concern in 70% (95% CI 65–74%) of these participants.

**Discussion:**

Psychosocial screening with HEARTSMAP-U can be reliably implemented in a post-secondary population as compared to a clinical clinician evaluation. Interestingly, a large cohort of respondents (10%) were deemed to have no psychiatric concerns by clinician evaluation, but severe concerns based on HEARTSMAP-U self-assessment. This specific population may represent a target group for future screening interventions.

## Introduction

The incidence of diagnosable mental health conditions and general psychological distress is rising among postsecondary students. In the United States, a study across 196 institutions (n = 155,026) reported a significant increase in lifetime mental health diagnoses—from 22% in 2007 to 36% in 2017—alongside a rise in treatment-seeking behavior from 19% to 34% over the same period [1]. Similarly, in Canada, self-reported diagnoses of anxiety, depression, addiction, eating disorders, bipolar disorder, and schizophrenia increased among postsecondary students between 2013 (n = 22,995) and 2019 (n = 38,127) [2]. The prevalence of mental health diagnoses within the past year rose from 22% to 37%, while formal treatment-seeking increased from 31% to 37% [2].

Despite the growing need, barriers such as limited access to care and prolonged wait times impede students from receiving timely support. Service saturation, complex referral processes, and fragmented care pathways contribute to these challenges and represent potential areas for system-level intervention [3].

The university environment plays a critical role in supporting the mental health of young adults. Canada’s National Standard for Post-Secondary Student Mental Health and Well-Being emphasizes the importance of mental health screening and early identification of unmet needs within campus health systems [4]. Universal screening and resource navigation interventions may help institutions identify and respond to a wide range of psychological (e.g., depression, anxiety) and social (e.g., housing, relationships) challenges—collectively referred to as psychosocial concerns [5]. Psychosocial health spans mental, emotional, and social dimensions and significantly impacts students’ well-being.

Screening tools can support self-awareness, improve mental health literacy, and help students assess their mental health status, which is especially important given that low perceived need often deters resource utilization [6]. Universal screening can ensure that all students, including those from equity-seeking groups (e.g., racialized, disabled, gender minority students), have equal opportunities for accessing supports. For instance, in a campus-wide initiative by Kodish et al. (2022) 73.3% of participants were from racially diverse backgrounds [7]. Although racialized students were less likely to have received previous treatment compared to Caucasian students, they were just as likely to initiate treatment post-screening.

However, implementation of screening tools within postsecondary health systems remains inconsistent due to several challenges. Firstly, positive screens can strain system capacity. Second, many screening tools were not developed in collaboration with students and may fail to reflect their unique priorities. For example, while the Counseling Center Assessment of Psychological Symptoms evaluates various mental health concerns, its relevance to students’ lived experiences remains unclear [8]. Additionally, many tools focus narrowly on specific mental health conditions, whereas multidimensional tools, such as our recently developed HEARTSMAP-U assess a broader range of student stressors.

Lastly, a critical shortcoming of existing tools is that many provide symptom screening without offering follow-up support, thus limiting their practical utility. The U.S. Preventive Services Task Force recommends depression screening only when adequate follow-up systems are in place [9]. Without such support, students may face difficulty accessing appropriate services. Although the integration of screening and support is promising, it remains understudied. HEARTSMAP-U addresses this gap by combining multi-domain assessment with supportive recommendations.

This study aims to evaluate the sensitivity and specificity of HEARTSMAP-U against clinician assessments, with particular emphasis on evaluating the help-seeking process, in hopes of establishing a scalable, universal screening tool for adolescent psychosocial well-being.

## Methods

Full ethics approval was received from the UBC Children’s and Women’s Research Ethics Board in 2022, which was reaffirmed annually through 2026. Written informed consent was obtained from each of the participants prior to their involvement.

We conducted a prospective validation study with assessments at two time points: baseline evaluation and a 3-month follow-up. Data collection occurred between December 2021 and April 2024. All procedures were administered remotely through individual Zoom sessions and included both self-report surveys and clinician-administered interviews. The clinician’s assessment consisted of their typical mental health intake evaluation. We allowed the two clinicians the freedom to perform this as per their usual practice to keep the process as close as we could to real world conditions. Following their intake assessment, each participant was accordingly assigned a perceived degree of need (no concern/mild/moderate/severe) in each of HEARTSMAP-U’s psychosocial domains. Clinicians trained together on numerous cases prior to commencement and remained blinded to participant self-assessments throughout the process. Apart from training together, our two clinicians did not overlap on participant cases, therefore inter-rater reliabilities could not be calculated. Participants received compensation in the form of gift cards or checks: CAD $60.00 (USD $45.40) at baseline and CAD $50.00 (USD $36.37) at follow-up.

Eligible participants were students aged 17 years and older, enrolled at either UBC Vancouver or UBC Okanagan. Exclusion criteria included: lack of access to a laptop or desktop computer; unavailability for the follow-up session within five days of the three-month mark; current or previous enrollment in the study; and residence outside British Columbia. Recruitment was conducted online with support from a student-led mental health advocacy organization.

Sampling was conducted in two stages:

- Stage 1: A convenience sample of 25 consecutively enrolled students.
- Stage 2: Quota-based purposive sampling to ensure demographic diversity across gender identity, racial identity, student type, and self-rated mental health status. Quotas were informed by UBC institutional data, the Canadian census, and literature on postsecondary mental health. Interested students completed a brief 1-minute survey to support quota-matching.

Sample size was determined a priori based on precision requirements for estimation of diagnostic accuracy parameters. The primary parameter of interest was sensitivity, which was assumed to be approximately 90% based on prior validation studies in the pediatric emergency department [10]. To achieve an absolute precision of ±6% around the sensitivity estimate at a 95% confidence level, approximately 100 participants with the condition of interest were required. This approach follows established recommendations for diagnostic validation studies, which emphasize ensuring an adequate number of affected individuals to obtain precise estimates of sensitivity and specificity [11].

Assuming a conservative prevalence of clinically significant mental health concerns of 20% in the target population [2], a total sample size of approximately 500 participants was required to yield the necessary number of positive cases.

### Measures

HEARTSMAP-U is a self-assessment tool developed to evaluate the psychosocial well-being of postsecondary students. It assesses ten key domains: Housing & Material Security, Education & Activities, Relationships, Thoughts & Anxiety, Substances & Behavioral Dependencies, Safety, Sexual Wellness, Mood, Abuse, and Professionals & Resources. This tool is adapted from a previously validated pediatric screening instrument— MyHEARTSMAP—with modifications to reflect the unique needs and experiences of postsecondary students. The adaptation process, described in detail elsewhere [12], was guided by student input and clinician expertise to ensure the tool remained age-appropriate, relevant, and clinically meaningful. In brief, this was a three-phase tool adaptation process, which included a cross-sectional mental health professional review, and an iterative series of focus groups to refine tool content and ensure relevance to users [12]. For example, the original “Home” section was redefined as “Housing & Material Security” to better capture the realities of students navigating independent living and financial challenges. The other sections of MyHEARTSMAP included education and activities, drugs and alcohol, relationships and bullying, thoughts and anxiety, safety, sexual health, mood, abuse, and professional resources – many of which were kept with minor adjustments for HEARTSMAP-U.

Each section contains a single item rated on a 4-point Likert scale ranging from 0 (no concern) to 3 (severe concern), accompanied by brief descriptors to help students select the most appropriate score. If students report any level of concern (e.g., score > 0), they are prompted to indicate whether they have accessed resources to address their concerns and are provided with an open-text box to describe their situation in more detail. This design supports both structured assessment and personalized reflection, offering insight into students’ psychosocial stressors while facilitating mental health literacy and help-seeking. Each of the 10 sections maps to one of 6 domains: Psychiatry, Abuse, Substance Use, Sexual Health, Education and Activities, and Housing.

Following the completion of the assessment, students were provided with curtailed recommendations for each domain, which were reflective of their degree of need. For example, a severe degree of concern may inform the participant to present to their nearest emergency department, while a mild concern may suggest relevant peer support groups.

### Statistical analyses

Upon completion of the baseline self-assessments and evaluation by a clinical counsellor, participant scores in each domain were available for analysis and direct comparison. Clinician and HEARTSMAP-U data were coded and stratified into risk groups where appropriate, including groups of “severe”, “moderate”, “mild”, and “no concern” (See supplement Data Coding).

Once stratified, participant recommendations from clinician and HEARTSMAP-U assessments were then analyzed by use of RStudio to compute sensitivities, specificities, and predictive values for each domain. Standard clinician intake assessments were used as the reference standard. 95% confidence intervals were calculated by way of the Clopper-Pearson binomial method in R. Of note, a discrepant cohort was identified early in the analysis, which was defined by a “severe” degree of mental health concern by the HEARTSMAP-U tool, but “no concern” on clinician evaluation. This group underwent further quantitative and qualitative analysis, which included review and thematic coding of participants’ narrative entries in the HEARTSMAP-U tool.

Given the high prevalence of mental health concerns, the tool was primarily created for psychiatric screening. As a result, the additional domains were analyzed less rigorously. Their low prevalence of concern was quickly identified as a limiting factor in performance, therefore much of the focus was placed on psychiatric performance.

## Results

**Fig 1:**
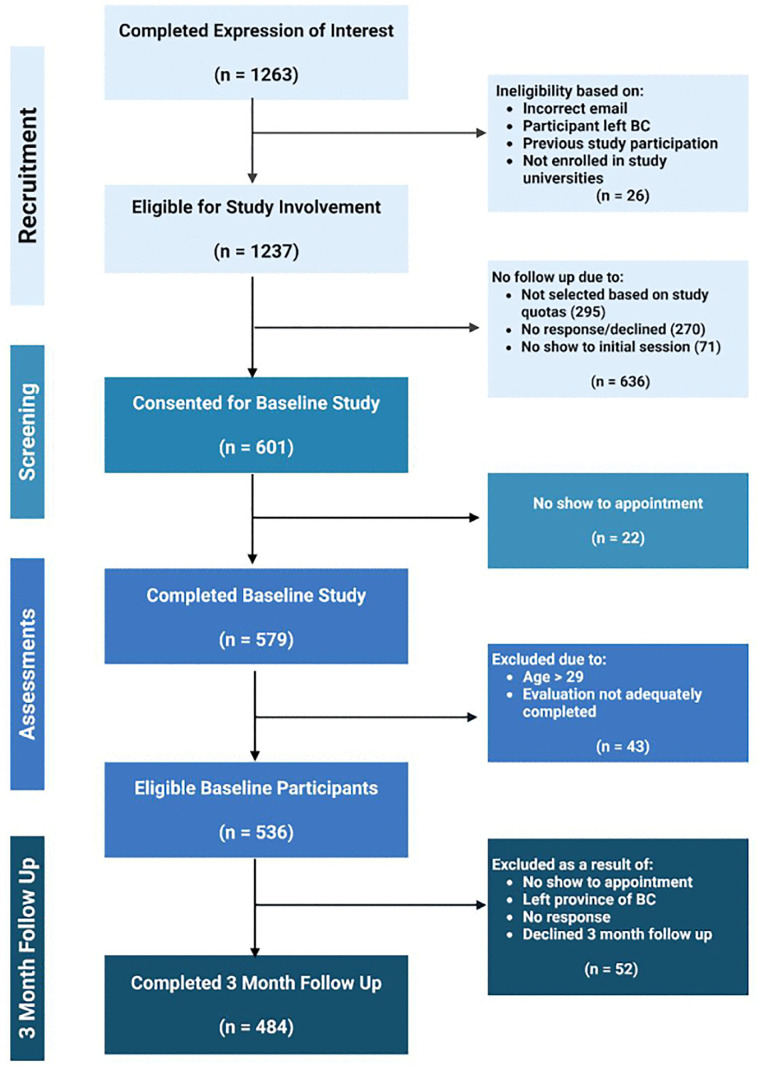
Study recruitment flow chart.

Between December 2021 and April 2024, 1263 university students >17 years of age expressed interest to enroll in our study at UBC Vancouver (86.4%) and UBC Okanagan (13.6%) in British Columbia. A total of 536 participants met the study’s eligibility criteria, consented for the baseline study, and completed both the self-assessments and clinician evaluation. Of these participants, 90.3% (484 of 536) were retained through the 3 month follow up process, allowing for re-evaluation, and the opportunity to provide feedback on their prior recommendations, along with any actions they took as a result. A flowchart of the study enrollment can be seen in Fig 1.

The recruitment process yielded a diverse group of study participants aged 17–29, with varying ethnicities, genders, and sexualities. This was generally in keeping with the local university demographics as a whole [13]. Study participants had a median age of 21.5 years, and 65.6% identified as female. A detailed breakdown of participant characteristics is detailed in Table 1.

**Table 1:** Baseline participant demographics.

Characteristics	Answers	Participants N (%)
**Total**		**536 (100%)**
**Age**		
	**17-20**	**220 (41.0%)**
	**21-25**	**257 (47.9%**
	**26-29**	**59 (11.0%)**
**Sex**		
	**Female**	**352 (65.6%)**
	**Male**	**177 (33.0%)**
	**Prefer Not to Answer**	**7 (1.3%)**
**Gender**		
	**Female**	**320 (59.7%)**
	**Male**	**177 (33.0%)**
	**Agender/Gender Fluid/Non-Binary/Two-Spirit**	**25 (4.7%)**
	**Unsure/Use a Different Term**	**11 (2.1%)**
	**Prefer Not to Answer**	**3 (0.6%)**
**Sexuality**		
	**Straight**	**342 (63.8%)**
	**Gay**	**40 (7.5%)**
	**Bisexual**	**101 (18.8%)**
	**Not Listed**	**29 (5.4%)**
	**Prefer Not to Answer**	**24 (4.5%)**
**Ethnicity**		
	**African**	**14 (2.6%)**
	**European**	**160 (29.9%)**
	**Middle Eastern**	**23 (4.3%)**
	**South or SE Asian**	**305 (56.9%)**
	**South American**	**13 (2.4%)**
	**Indigenous**	**10 (1.9%)**
	**Not Listed/Prefer Not to Answer**	**11 (2.1%)**
**Campus**		
	**UBC-Okanagan**	**73 (13.6%)**
	**UBC-Vancouver**	**463 (86.4%)**

On baseline evaluation, the sensitivity of HEARTSMAP-U self-assessments in identifying the presence of issues ranged from 100% (95% CI 74–100%) in the abuse domain, to 57% (95% CI 18–90%) in the sexual health domain. The specificity of participant assessments in identifying the absence of issues ranged from 93% (95% CI 91–95%) in the substance use domain, to 41% (95% CI 36–45%) in the education domain. Detailed utility measures for each domain in the baseline study are reported in Table 2. Sensitivity can be defined as the tool’s ability to detect an individual’s unmet need as compared to their evaluation by a mental health clinician intake evaluation. This includes mild, moderate, and severe degrees of concern for each evaluation.

**Table 2:** HEARTSMAP-U baseline assessment data, including sensitivity, specificity, and predictive values as compared to clinician assessment.

	Mental Health	Substance Use	Abuse	Sexual Health	Education/ Activities	Housing
**Sensitivity**	**90% (83-94%)**	**64%** **(31-89%)**	**100% (74-100%)**	**57%** **(18-90%)**	**82%** **(69-91%)**	**80%** **44-97%)**
**Specificity**	**54% (49-59%)**	**93%** **(91-95%)**	**58%** **(54-62%)**	**87%** **(84-90%)**	**41%** **(36-45%)**	**64%** **(60-68%)**
**PPV**	**40% (35-46%)**	**17%** **(7-31%)**	**5%** **(3-9%)**	**5%** **(2-13%)**	**12%** **(9-16%)**	**4%** **(2-8%)**
**NPV**	**94% (90-97%)**	**99%** **(98-100%)**	**100%** **(99-100%)**	**99%** **(98-100%)**	**96%** **(92-98%)**	**99%** **(98-100%)**

In the psychiatric domain, which represented the main area of interest, there was a sensitivity of 90% (95% CI 83–94%) in detecting mental health concerns, and a specificity of 54% (95% CI 49–59%) in identifying an absence of concerns in the baseline study. On the follow up iteration at 3 months, participant assessments demonstrated a sensitivity of 79% (95% CI 67–88%) in identifying psychiatric concerns, with a specificity of 41% (95% CI 36–46%) in discerning a lack of psychiatric concerns.

A distinct cohort of participants emerged on review of baseline screening data, which was composed of students deemed to have “severe” mental health concerns on their self-assessments, but “no concern” when assessed by clinical counselors. On the baseline survey, this cohort represented 9.1% of the study group, with 49 of 536 participants fitting these criteria. In the 3 month follow up study, this cohort was essentially unchanged in size, 43 of 484 participants—representing 8.9% of the follow-up study group. Detailed utility measures for each domain in the three month follow up study are reported in Table 3.

**Table 3:** HEARTSMAP-U 3-month follow-up assessment data, including sensitivity, specificity, and predictive values as compared to clinician assessment.

	Mental Health	Substance Use	Abuse	Sexual Health	Education/ Activities	Housing
**Sensitivity**	**79% (67-88%)**	**50%** **(1-99%)**	**100% (40-100%)**	**N/A**	**100%** **(78-100%)**	**50%** **(7-93%)**
**Specificity**	**41% (36-46%)**	**93%** **(91-95%)**	**61%** **(56-65%)**	**88%** **(85-91%)**	**46%** **(42-51%)**	**68%** **(63-72%)**
**PPV**	**16% (12-21%)**	**3%** **(0-16%)**	**2%** **(1-5%)**	**0%** **(0-16%)**	**6%** **(3-9%)**	**1%** **(0-4%)**
**NPV**	**93% (88-96%)**	**100%** **(99-100%)**	**100%** **(99-100%)**	**100%** **(99-100%)**	**100%** **(98-100%)**	**99%** **(98-100%)**

**Fig 2:**
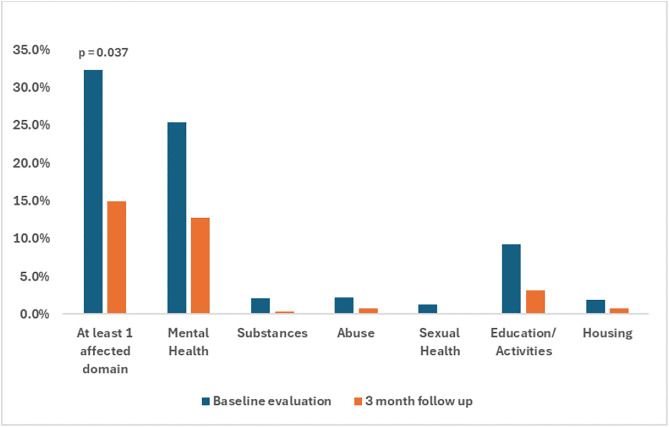
Prevalence of participants’ unmet needs in each psychosocial domain at baseline and 3 month follow up as assessed by clinical counsellor.

Overall, screening by clinical counsellors identified 32.3% of participants as having issues in at least one psychosocial domain, which were accordingly given recommendations by the HEARTSMAP-U tool. When re-evaluated at their 3 month follow up, only 14.9% were found to have concerns in one or more of these domains on clinician assessment. This included a 12.6% overall reduction in participants with a mental health concern (49.6% relative reduction), which is displayed in Fig 2.

**Fig 3:**
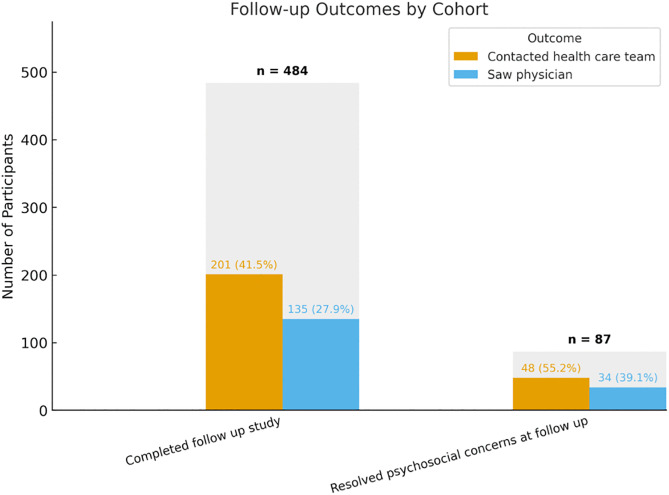
HEARTSMAP-U help-seeking process undertaken by participants at 3-month follow-up: comparing all participants to those who improved from some degree of psychosocial concerns to “no concerns” based on clinician assessment.

Of note, 201 of the 484 participants in the 3 month follow up (41.5%) stated that their baseline study recommendation prompted them to reach out to their health care team, with 135 of them (27.9%) seeing a medical practitioner regarding these concerns. We also found that of the 87 individuals who had some degree of psychiatric concern at baseline but improved to “no concern” at 3 month follow up, 55.1% either attempted to reach out or managed to see their health care team in that interim period. This help-seeking process can be seen in Fig 3.

## Discussion

Our study indicates that young adults can self-identify psychiatric concerns with a sensitivity comparable to similar previous studies [10]. For instance, the sensitivity for detecting mental health concerns was 90% (95% CI 83–94%), demonstrating that participants could recognize and report distressing symptoms, which were then met with corresponding recommendations. These findings suggest that self-assessment tools like HEARTSMAP-U can serve as effective screening mechanisms for identifying individuals who may require further support. This aligns with literature indicating that young adults are increasingly willing to engage with self-guided mental health resources, especially in digital formats [14]. Given the growing prevalence of mental health concerns among university students [15], HEARTSMAP-U presents an opportunity to provide a scalable and accessible screening tool that can facilitate early intervention.

MyHEARTSMAP was a predecessor study conducted on youth in the emergency departments of BC and Alberta in 2019 [10]. This study demonstrated high sensitivity (92.7% for youth, 93.1% for guardians) and specificity (98.5% for youth, 98.9% for guardians) in identifying psychiatric concerns. Compared to MyHEARTSMAP, HEARTSMAP-U exhibited more variable performance across domains. For instance, the sensitivity for detecting any level of mental health concerns was comparable (90% (95% CI 83–94%) for HEARTSMAP-U vs. 93.1% (95% CI 89.5–95.8%) for MyHEARTSMAP). However, specificity for psychiatric concerns was notably lower in HEARTSMAP-U (70% (95% CI 65–74%)) compared to MyHEARTSMAP (98.5%), indicating a higher likelihood of false positives. Notably, several domains were seen to be quite specific on HEARTSMAP-U self-assessments, such as substance use and sexual health, which had specificities of 93% (95% CI 91–95%) and 87% (95% CI 84–90%), respectively. This is potentially attributable to how low the true prevalence of concern was in these domains, which ranged from 1–2% in the substance use and sexual health domains, but approximately 25% in the psychiatric domain.

The overall low prevalence of concerns in all domains except for mental health led to quite significant variability in performance. As such, HEARTSMAP-U is reasonably positioned to assess the mental health domain, with the prevalence of psychiatric concerns serving as a better target for universal screening. There may be some utility in the educational/activities domain, given its 10% prevalence of concerns in our cohort. The wide variance in the sensitivity and specificity of the other domains demonstrated the limited utility of HEARTSMAP-U in screening for rare concerns.

We see that a staggering 201 of the 484 participants (41.5%) who completed the 3 month survey stated that because of their baseline recommendations, they reached out to their health care team, with 135 of these 201 seeing a physician in this interim period. This was reflected by a 12.6% decrease in prevalence of mental health concerns (25.4 to 12.8%) on repeat clinician evaluation at 3 months, with 87 participants improving from at least mild concerns to “no concerns”. This reduction may be due to numerous factors, such as seasonal variation, spontaneous resolution, or slight variations in how clinician’s evaluated participants at follow up. We have identified this as an area for future research to determine the true effect of our universal screening on the help-seeking process and the potential alleviation of mental health burden. Of note, the prevalence of mental health concerns and relative participant engagement may be somewhat inflated given our recruitment was done in part through the university’s mental health advocacy network.

Despite its strong sensitivity, there was notable variation between HEARTSMAP-U self-assessments and clinician evaluations, which may be attributable to several factors. Firstly, some participants may have been more comfortable reporting mental health concerns in a digital format rather than during a face-to-face assessment. This is consistent with research suggesting that youth prefer to disclose information to digital tools rather than directly to clinicians, especially on topics deemed embarrassing such as sexual health or substance use, two domains found with lower specificity in the current study [14]. Additionally, clinicians may have accounted for contextual factors, such as whether a participant was already receiving care, that the self-assessment tool could not adjust for, leading to differences in classification (i.e., if the participant reported receiving effective care, the severity was downgraded). Furthermore, HEARTSMAP-U was designed to err on the side of caution, increasing its sensitivity to ensure that no severely unwell participants were missed, even at the expense of specificity. Relatedly, some participants may not disclose mental health concerns to any screening tool, whether digital or face-to-face. In fact, in one study up to 18.1% of youth of a comparable demographic who had suicidal thoughts did not disclose this to a mental health clinician despite having adequate access to care [16]. Increasing research on “non-labelers” identifies a cohort of people who have symptoms of poor mental health, but do not endorse these symptoms due to fear of stigma [17].

Given that the self-assessments and clinician evaluations were conducted virtually, it is important to recognize that this may have also been a limitation for some of the participants. While it is becoming increasingly common for health care to be delivered virtually today, some participants would likely feel more comfortable disclosing sensitive information in a face-to-face setting after adequate rapport had been established. However, given HEARTSMAP-U’s design as an accessible, virtual tool, the remote nature of data collection was likely representative of its real-world use, and we hope it would serve as a gateway to establish real-world connections.

### Non-labelers cohort

Interestingly, a large cohort of respondents (9.1% of the study group) were deemed to have no psychiatric concerns by clinician evaluation, but disclosed information on their HEARTSMAP-U self-assessment in keeping with a severe degree of need. This discrepant cohort can be described as “non-labelers”, and serve as a target group for intervention, as they may not be adequately picked up by existing measures. Some of this discrepancy may be attributable to the nuance in evaluating a participant’s current available supports (therefore clinicians would not suggest further action or deem a participant high risk), though we believe a significant portion is due to the comfort in interacting with a digital tool.

In the qualitative portion HEARTSMAP-U where participants elaborated on their experiences, these individuals often minimized their symptoms or described confusion on if support would be effective. This group often questioned whether they needed help compared to others, and highlighted their own ability to manage without support, which provided important insights into this group’s mentality. Some specific quotes attributable to this discrepant cohort are as follows:

“I tend to have suicidal thoughts during panic attacks, though I never seriously plan or consider taking any real action...I feel down most of the time for no reason, especially when I’m alone...I went to one therapy session and never followed up for continuous sessions... I am unsure if I really need mental health support.”

“I don’t have suicidal thoughts and do not engage in self-harm, but wonder how life would be for others without me there...Recently, I have been having mood swings such as anger or sadness...I don’t think accessing support is worth it for me because I feel as if I am just in a slump. I am also not willing to do so financially as well”

“Definitely haven’t been taking care of myself, laundry, cleaning up, cooking regularly, financially, standard student things...[I] have accessed it [counselling] in the past, but UBCO [University support] is overworked, and I can’t get help as quickly as i need it when I do, or feel like I may be holding things up for someone who may need it more, and I ought to wait until it’s actually important”

Though this group is small, the demographics align with current research (predominantly male, young 19–20, racial minority) which explores those who do not self-label or seek treatment [16]. Since this analysis is based on those who did write more, we assume that there is an even higher incidence in the current study of participants who have similar experiences and beliefs about their mental health but chose not to write more in the qualitative portion. Estimates from other research put this group at about 20–30% [17], whereas this group represents 9.1% of our data set. Recognizing non-labelers as an important population, as well as the reasons behind non-endorsement of mental illness (e.g., stigma), may explain why this cohort did not disclose these symptoms to clinicians or self-identify a need for help seeking.

Future research should further analyze the HEARTSMAP-U long-term effectiveness in young adult populations. One possible study includes longitudinal monitoring, where students complete several follow-up evaluations to assess changes in their mental health status over time, allowing for a better understanding of symptom persistence. Other possible studies could expand the population of interest beyond university students, including young adults in different settings, for example, high schools, the workforce, and community health clinics. Implementing HEARTSMAP-U in a non-academic setting could potentially support its adaptation for wider use. It is imperative to ensure the data collected from young adults in the context of mental health status remains secure and protected. As with any digital mental health tool, and the sensitivity regarding the information collected, clear policies on data storage, access, and confidentiality must be established to prevent misuse. Potential ethical dilemmas may arise if a user self-reports a serious concern like suicidal ideation, thus, future studies should analyze if implementing automated crisis support features is possible within HEARTSMAP-U.

## Conclusions

In conclusion, the use of HEARTSMAP-U self-assessments in a university population identified psychiatric concerns with a high sensitivity, however there was variable sensitivity and specificity across other domains. Overall, the tool performed well in the mental health domain, but the low prevalence of unmet need across the additional domains (Abuse, Substance Use, Sexual Health, Education and Activities, and Housing) limited the performance of HEARTSMAP-U. This serves as a potential topic for further research and refinement in order to effectively screen for issues in these areas.

There are several key approaches to maximize the effectiveness of HEARTSMAP-U. One key approach is to integrate HEARTSMAP-U into the university’s existing student wellness programs. This could be implemented through screenings at key academic steps such as enrollments, between-term wellness assessments, and annual check-ups. Raising awareness among both staff and students could potentially help create a network of support, making it more accessible for students to use the tool when needed. Additionally, educational initiatives like workshops and meetings on self-monitoring mental health digital tools could further increase their utility [18]. Given the high rate of false positives, the tool would likely function best when paired with an element of human screening validation, particularly for individuals identified as high risk. We envision this being built into the current mental health support waitlisting: ensuring those who need help most are seen expeditiously.

**Key Practitioner Message**

- Our study is based upon current data regarding young adult mental health, specifically in Canada. Two predecessor screening tools (SASS and CHQ) were designed to capture similar elements of adolescent mental health, but this novel tool was developed to assess unique and pointed areas of psychosocial interest.
- The HEARTSMAP-U self-assessment tool demonstrates high sensitivity in identifying student psychosocial concerns, which aids early intervention. Its integration into university wellness programs, coupled with human validation, could address rising student mental health needs. This scalable approach, which empowers self-screening and facilitates resource access, offers a significant contribution to improving postsecondary mental health support.

## Supporting information

S1 Fig HEARTSMAP-U tool sections and relevant tool recommendations. (DOCX)

S1 Table Data coding. (DOCX)
