# Supplementary material for: Utility of HEARTSMAP-U for psychosocial screening and mental health resource navigation in the young adult population
Source: PLoS One. 2026 Jul 13;21(7):e0353390. doi: 10.1371/journal.pone.0353390 (PMC13362112; doi:10.1371/journal.pone.0353390)
Supplement: S1 Fig — (DOCX) [file pone.0353390.s001.docx]

**S1 Fig.** **HEARTSMAP-U Tool Sections and Relevant Tool Recommendations**

**
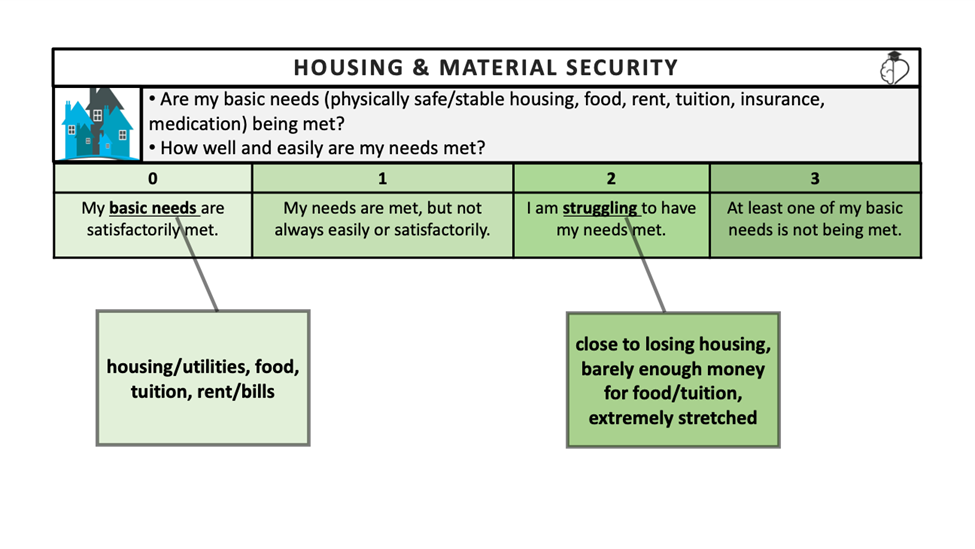
**

| **HOUSING AND MATERIAL SECURITY RECOMMENDATIONS** |  |
| --- | --- |
| We are sorry to hear that you are having trouble meeting your basic needs. We suggest you try to seek support by meeting with a **student service professional** or accessing **community-based support services**. |  |
|  |  |
| It seems like you are managing to meet your basic needs without problems at the moment. If in the future you feel like this has changed, you can come back and redo this assessment so that we can provide you with some more options for support. You can also check out the **UBC Resources** pages below for more information of financial planning, scholarships, and student job opportunities.    ● *Receiving this prompt indicates that you did not receive a recommendation for this domain.* |  |


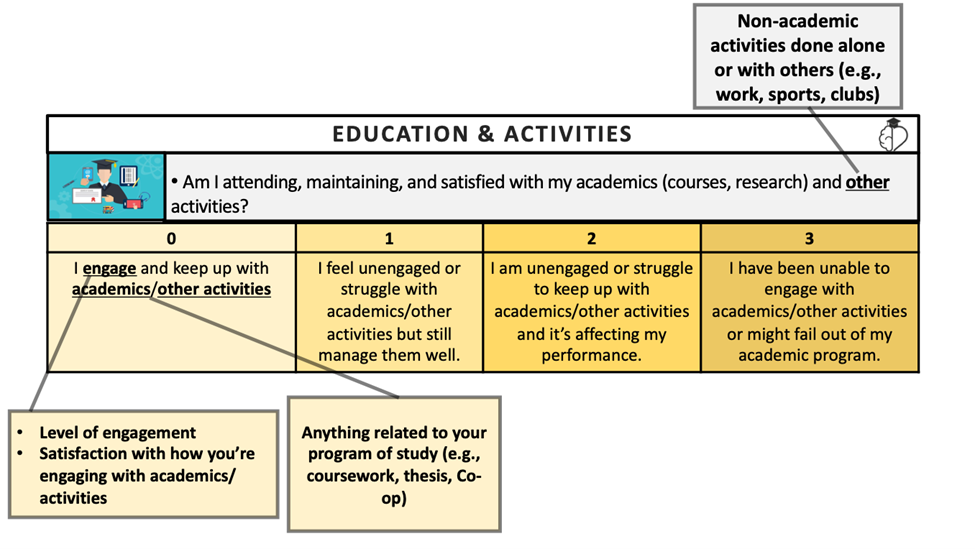


| **EDUCATION AND ACTIVITIES** |
| --- |
| For any challenges related to education, we recommend you talk to an **academic advisor** in your faculty/department about concerns related to your program, an **accessibility advisor** to discuss helpful concessions or accommodations, or an **ombudsperson** to discuss potential issues with the learning environment. You can also chat with a **peer academic coach** for helpful time management or study tips. |
| For any challenges related to non-academic activities or getting more involved at UBC, we recommend you chat with a trained **peer supporter** for tips on how to build connections at university. |
| You seem to be managing well with your education and other activities. If in the future you feel like this has changed, you can redo this assessment so that we can provide you with some more options for support.    ● *Receiving this prompt indicates that you did not receive a recommendation for this domain.* |


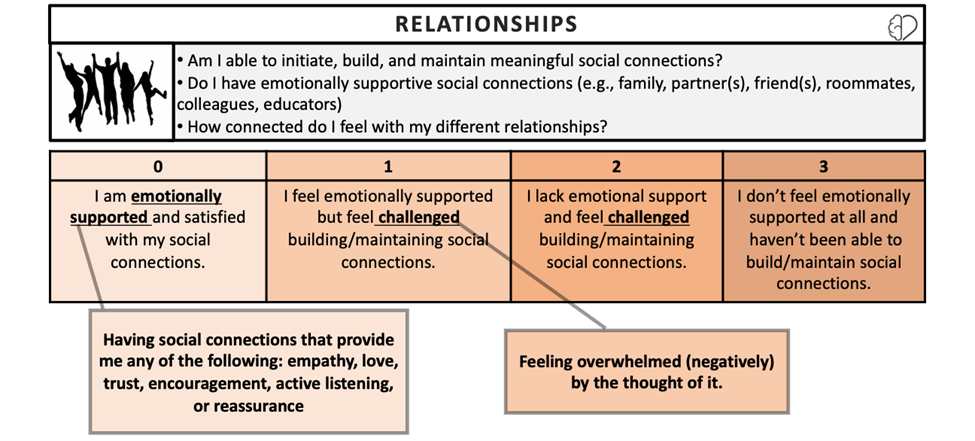


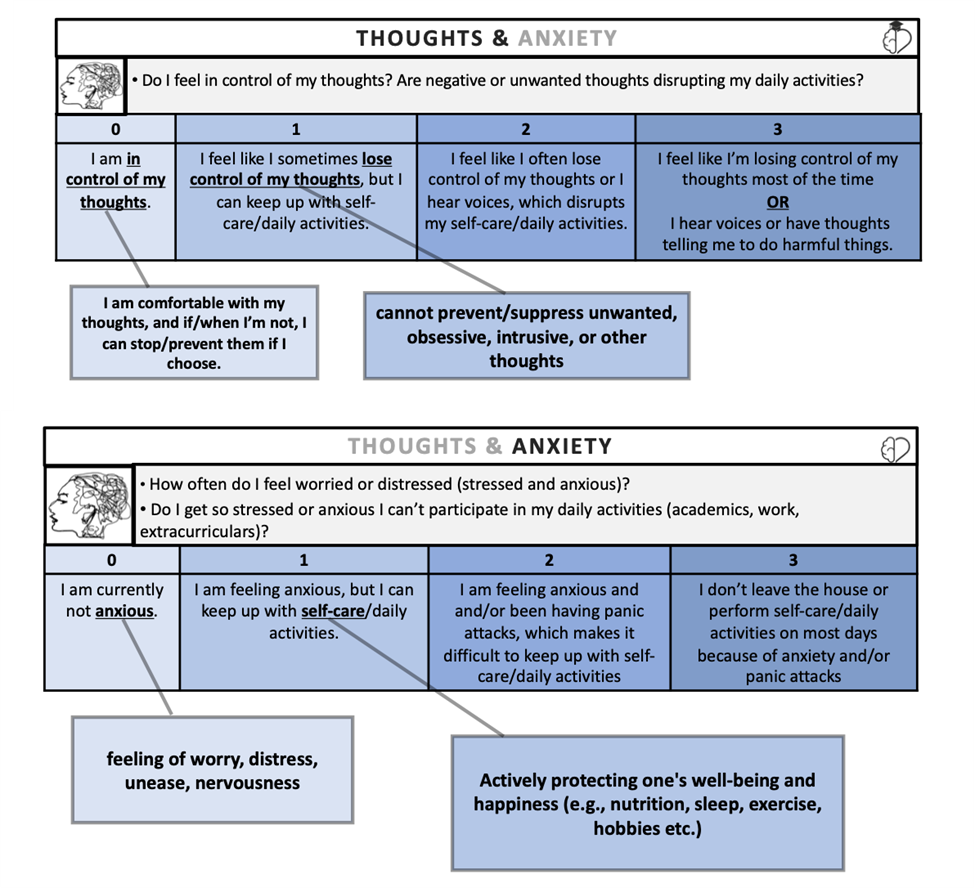


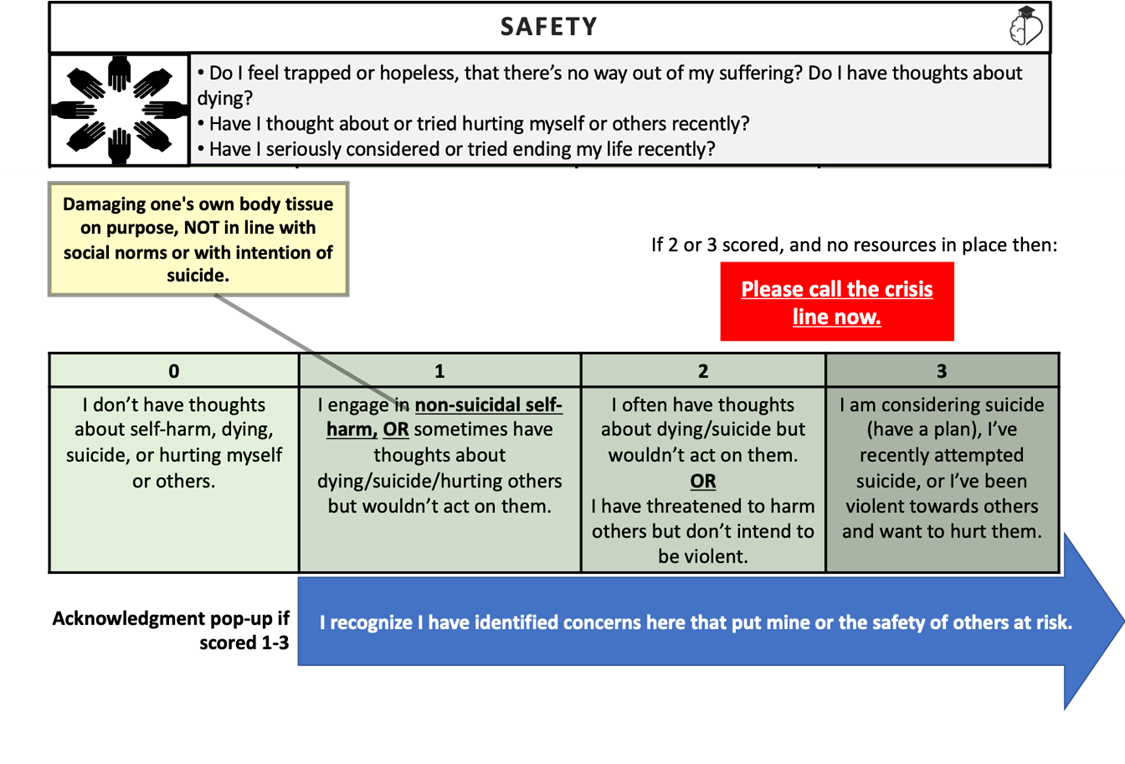


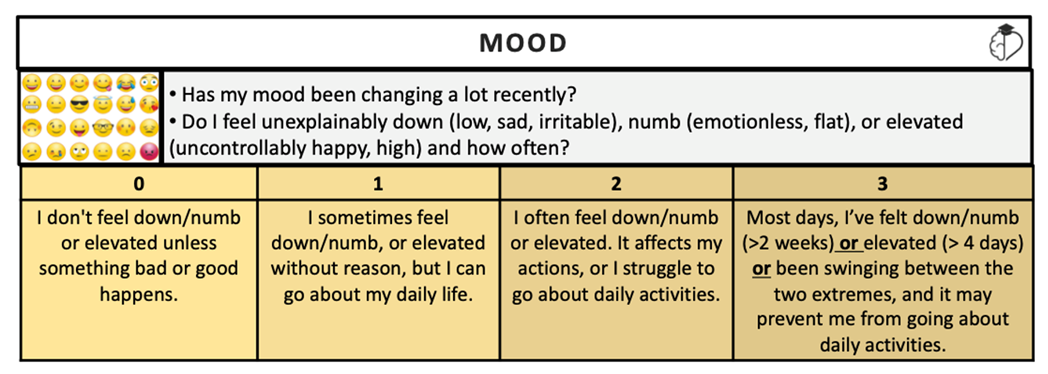


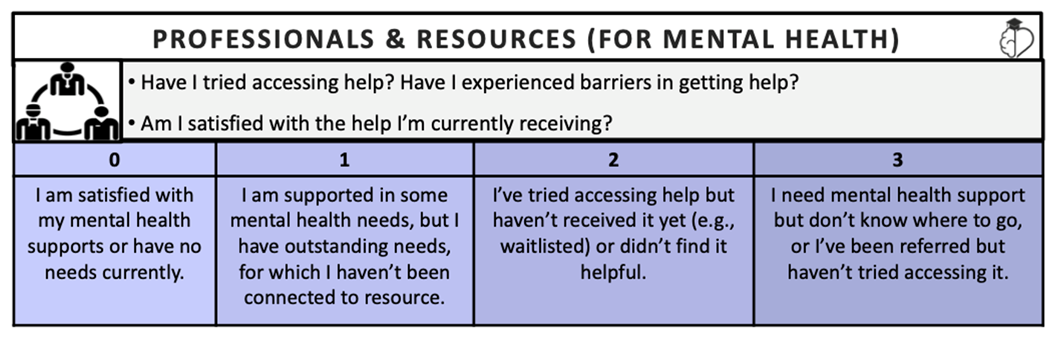


| **MENTAL HEALTH AND RELATIONSHIPS RECOMMENDATIONS** |  |
| --- | --- |
| Given that your concerns sound urgent, please consider seeking help as soon as possible to keep you safe. You can visit your nearest **emergency department**, contact a **crisis line** anonymously, or call 911 to receive support. We also recommend that to secure longer term support you make a same day appointment with a **primary care provider** and/or visit **counselling services**. |  |
| It sounds like you are experiencing some challenges that are having a large impact on your wellbeing. We recommend you seek help as soon as possible by making a same day appointment with a **primary care provider** (e.g., your family doctor) and/or **counselling services** so that they can support you through this. In the meantime, you can also contact a **crisis line** 24/7 or visit a **rapid access mental health clinic**. |  |
|  |  |
| University can be a stressful environment and many students go through difficult times. We recommend you make an appointment with a **counsellor** and/or a **primary care provider** (e.g., your family doctor) to discuss what you are going through and resources that are available to support you. In the meantime, you can also contact a **mental health support line** 24/7. |  |
|  |  |
| University can be a stressful environment and many students go through difficult times. We recommend you make an appointment with your family doctor or another **primary care provider** to discuss what you are going through and resources that may be available to support you. In the meantime, you can also chat with a **peer supporter** or contact a **mental health support line** 24/7. |  |
|  |  |
| Forming lasting relationships can be difficult, but there are resources available to help you. We recommend you make an appointment with a **counsellor** to talk about this and any other concerns you may have. In the meantime, you can also chat with a **peer supporter** or contact a **mental health support line** 24/7. |  |
|  |  |
| University can be a difficult time and many students experience mental health challenges at some point. We recommend you make an appointment with your family doctor or another **primary care provider** to discuss what you are going through and resources that may be available to support you. In the meantime, you can also chat with a **peer supporter** or contact a **mental health support line** 24/7. |  |
|  |  |
| It is normal to feel overwhelmed at times, especially in a stressful university environment, but there are people ready to listen and support you through this. We recommend that you chat with a trained **peer supporter** to get tips to manage these challenges and to talk through any problems you may be facing. In the meantime, you can also contact a **mental health support line** 24/7 and check out some of the **online resources** below. |  |
|  |  |
| Forming relationships can be difficult, especially in a large environment like a university, but there are people ready to listen and support you through this. We recommend that you chat with a trained **peer supporter** to get tips for building social connections or any other challenges you may be facing. You can also contact a **mental health support line** 24/7. |  |
|  |  |
| You appear to be managing well in terms of your mental health and relationships. If, in the future, you feel like this has changed, you can redo this assessment so that we can provide you with some more options for support. If you would still like to see available supports or learn more about **maintaining your wellbeing**, you can check out some of the resources below. If you do not currently have a primary care provider (e.g., your family doctor), it may also be useful to secure one in case you ever have additional needs in the future.  ● *Receiving this prompt indicates that you did not receive a recommendation for this domain.* |  |
|  |  |


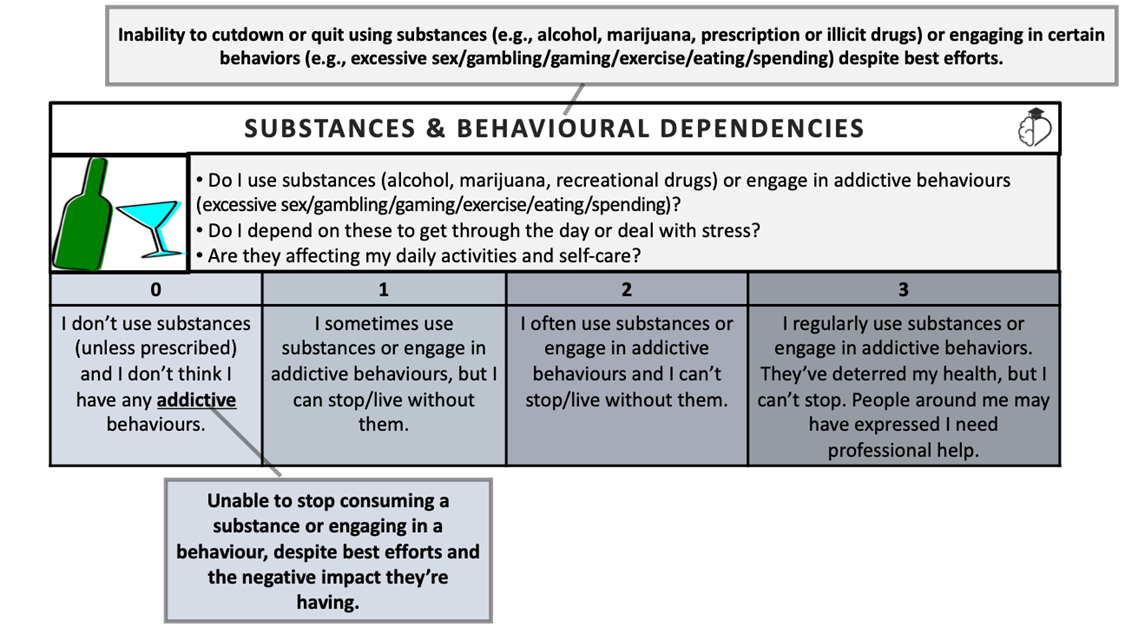


| **SUBSTANCE USE AND BEHAVIORAL DEPENDENCIES RECOMMNEDATIONS** |  |  |
| --- | --- | --- |
| Dealing with substance use or behavioural dependencies can be challenging, but you don't have to do it alone. We recommend that you consult a **primary care provider** (e.g., your family doctor) and explore some **online resources** to support you through this. For more immediate support, you can visit a **rapid access mental health clinic** or contact a 24/7 **support line**. |  |  |
|  |  |  |
| You appear to be managing well in terms of your substance use and behavioural dependencies. If, in the future, you feel like this has changed, you can redo this assessment so that we can provide you with some more options for support. If you would still like to see some resources, you can scroll through some **options below**. If you do not currently have a primary care provider, it may also be useful to secure one in case you ever have additional needs in the future.  ● *Receiving this prompt indicates that you did not receive a recommendation for this domain.* |  |  |
|  |  |  |


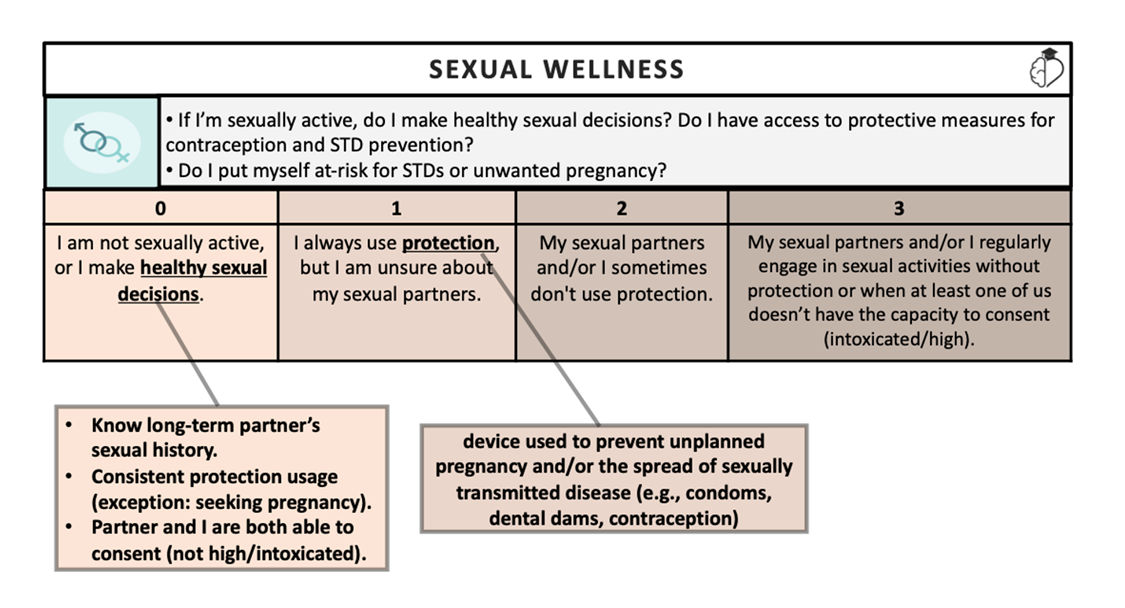


| **SEXUAL WELLNESS** |  |
| --- | --- |
| For your sexual-wellbeing, we recommend you talk to a **primary care provider** to learn more about safer sexual health practices. You can also check out some of the following **resources** to get more information. |  |
| We encourage you to communicate with your sexual partners about any concerns you may have. If you would like to learn more about sexual health, you can visit some of the **resources** below. |  |
|  |  |
| You do not seem to have any current concerns related to your sexual wellness. If you would like to learn more about sexual health, you can visit some **online resources** below. If you do not currently have a primary care provider, it may also be useful to secure one in case you ever have additional needs in the future.  ● *Receiving this prompt indicates that you did not receive a recommendation for this domain.* |  |


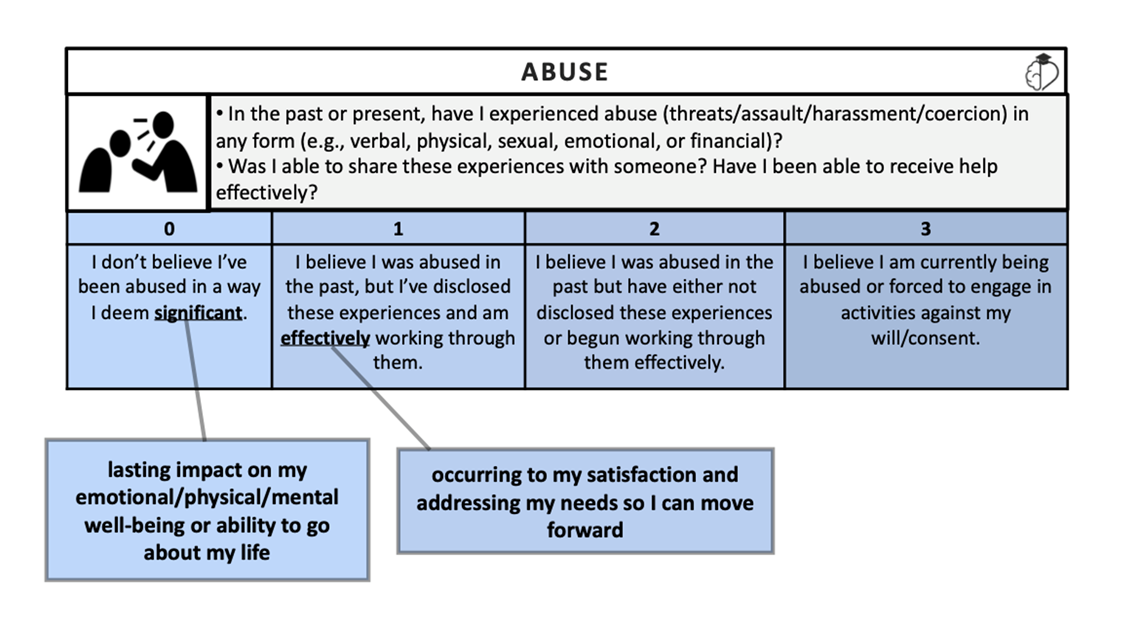


| **ABUSE** |
| --- |
| It seems like you are currently in an abusive situation and we want to make sure that you are safe and get the support you need. We recommend you talk to a **primary care provider**, trained **support worker**, or contact a **confidential support line** as soon as possible. You can also find out more information about what help may look like for each of these resources from their websites below. |
| It sounds like you have experienced abuse in the past. If and when you are ready, there are many resources available to support you both **in person** or **by phone**. You can also visit the following **websites** to find out more information about the services that these resources provide and what help may look like. |
| It sounds like you have experienced abuse in the past, but you have been able to access support and are working through this. If you would ever like additional support you can view available **resources** below. |
| You have no recommendations at this time. If in the future you feel like this has changed, you can redo this assessment so that we can provide you with some more options for support.  ● *Receiving this prompt indicates that you did not receive a recommendation for this domain.* |
