# Supplementary figures and images for: Utility of HEARTSMAP-U for psychosocial screening and mental health resource navigation in the young adult population
Source: PLoS One. 2026 Jul 13;21(7):e0353390. doi: 10.1371/journal.pone.0353390 (PMC13362112; doi:10.1371/journal.pone.0353390)

**S1 Table. Data Coding**


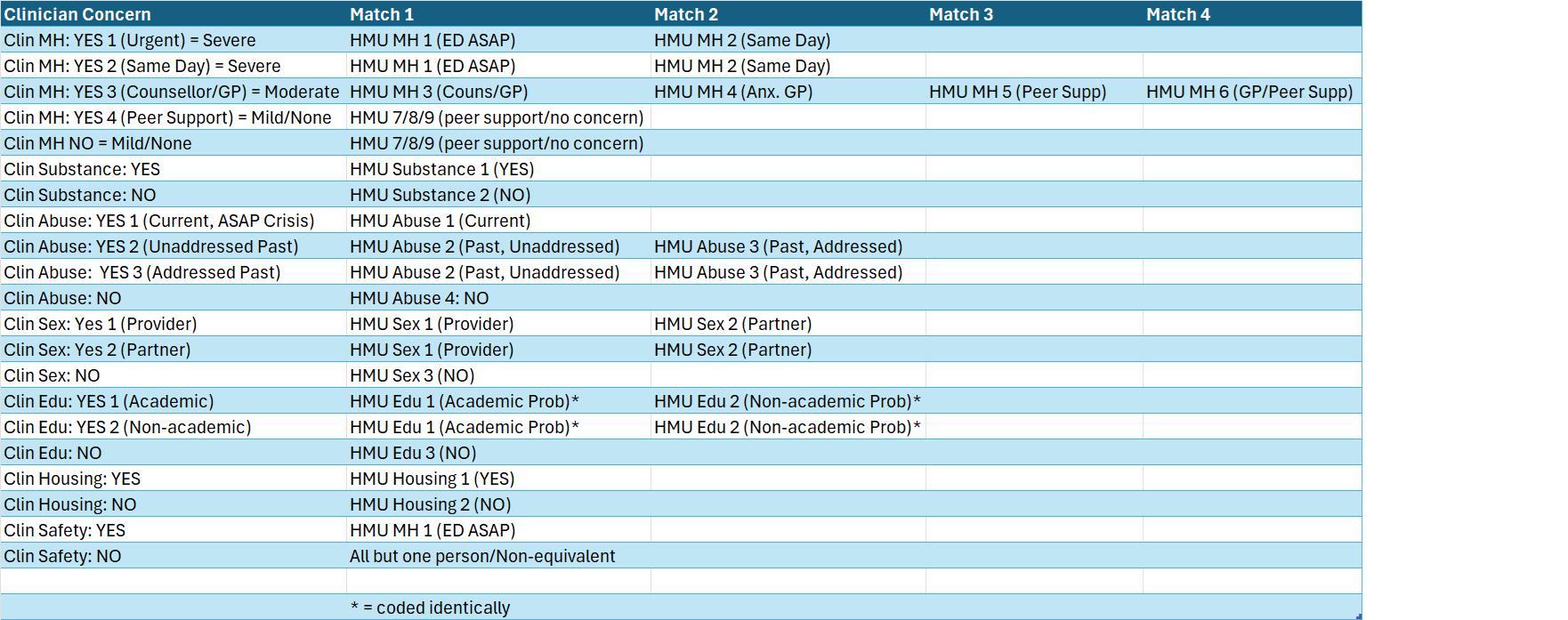

Supplement: S1 Table — (DOCX) [file pone.0353390.s002.docx]
