# Supplementary material for: Utility of HEARTSMAP-U for psychosocial screening and mental health resource navigation in the young adult population
Source: PLoS One. 2026 Jul 13;21(7):e0353390. doi: 10.1371/journal.pone.0353390 (PMC13362112; doi:10.1371/journal.pone.0353390)
Supplement: Attachment — Submitted filename: Responses to Reviewers.docx [file pone.0353390.s004.docx]

Responses to Reviewer’s Comments

Manuscript Reference Number: PONE-D-25-63524R1

**Utility of HEARTSMAP-U for psychosocial screening and mental health resource navigation in the young adult population**

Dear Dr. Souparno Mitra,

We are grateful to the reviewers and for your suggested edits and comments for improving the manuscript. We have made the suggested formatting changes as per your advice.

We have addressed all the reviewers’ comments and modified the manuscript as detailed below.

Thank you in advance.

**Editor Comments:**

1.Please ensure that your manuscript meets PLOS ONE's style requirements, including those for file naming. The PLOS ONE style templates can be found at
<https://journals.plos.org/plosone/s/file?id=wjVg/PLOSOne_formatting_sample_main_body.pdf> and <https://journals.plos.org/plosone/s/file?id=ba62/PLOSOne_formatting_sample_title_authors_affiliations.pdf>

Response: I have gone through each of these documents and meticulously corrected the formatting.

2. We note that you have indicated that there are restrictions to data sharing for this study. PLOS only allows data to be available upon request if there are legal or ethical restrictions on sharing data publicly. For more information on unacceptable data access restrictions, please see <http://journals.plos.org/plosone/s/data-availability#loc-unacceptable-data-access-restrictions>.
> Before we proceed with your manuscript, please address the following prompts:
> a) If there are ethical or legal restrictions on sharing a de-identified data set, please explain them in detail (e.g., data contain potentially identifying or sensitive patient information, data are owned by a third-party organization, etc.) and who has imposed them (e.g., a Research Ethics Committee or Institutional Review Board, etc.). Please also provide contact information for a data access committee, ethics committee, or other institutional body to which data requests may be sent.
> b) If there are no restrictions, please upload the minimal anonymized data set necessary to replicate your study findings to a stable, public repository and provide us with the relevant URLs, DOIs, or accession numbers. For a list of recommended repositories, please see
 <https://journals.plos.org/plosone/s/recommended-repositories>. You also have the option of uploading the data as Supporting Information files, but we would recommend depositing data directly to a data repository if possible.
 We will update your Data Availability statement on your behalf to reflect the information you provide.

Response: I have uploaded our data to a suggested repository and have made this available. It is referenced at the end of the manuscript and the url is included in the revised submission.

3. Please amend either the title on the online submission form (via Edit Submission) or the title in the manuscript so that they are identical.

Response: These are now identical.

4. Your ethics statement should only appear in the Methods section of your manuscript. If your ethics statement is written in any section besides the Methods, please move it to the Methods section and delete it from any other section. Please ensure that your ethics statement is included in your manuscript, as the ethics statement entered into the online submission form will not be published alongside your manuscript.

Response: The ethics statement is now included in the methods section and deleted from the end of the manuscript.

5. Please remove your figures from within your manuscript file, leaving only the individual TIFF/EPS image files, uploaded separately. These will be automatically included in the reviewers’ PDF.

Response: Figures have been removed from the manuscript file and have been uploaded separately.

6. Please include your tables as part of your main manuscript and remove the individual files. Please note that supplementary tables (should remain/ be uploaded) as separate "supporting information" files.

Response: Individual table files have been removed.

7. If the reviewer comments include a recommendation to cite specific previously published works, please review and evaluate these publications to determine whether they are relevant and should be cited. There is no requirement to cite these works unless the editor has indicated otherwise.

Response: Citations have been edited accordingly.

**Reviewer’s Comments**

**Reviewer #1:**

1. My primary concern with this study is conceptual. The study rationale and hypothesis are based on promotion of dichotomized mental health. Reading the abstract and introduction it feels like I will be reading about tactics for identifying the “mentally ill.” I put a great deal of effort into destigmatizing mental health, and a key component is to always address mental health on its continuum.

Response: We fully agree with you that we need to destigmatize mental health issues. The intention of the tool is to be a universal screening to check in rather than a dichotomous diagnostic tool. To ensure that the tool is reliable prior to launch, we have to assure there are no issues, false positives to drain the resource system, or blind spots. Our goal is to create a universal screening tool to help youth feel more comfortable with checking in on their mental wellness without needing an appointment or intake assessment. We have re-worded the purpose (last two lines) and discussion sections of the abstract to better reflect the intent of validating our screening tool. We have also made edits to the third paragraph of the introduction to put less emphasis on “identifying” and more towards supporting individuals, as we see your point regarding this dichotomizing language.

The psychometric evaluation of the tool has to evaluate what should necessitate further intervention. That is why we provide sensitivity and specificity against an intake assessment, that may or may not direct the user to further diagnostic evaluations.

2. As I read through the tool, it looks like a self-awareness tool paired with guidance to resources. That is something altogether different from what seems to be described in the Abstract and Introduction. I suggest you move away from the framework of tools for diagnosis (dichotomization) and move towards tools for mental health promotion and support.

Response: As mentioned above, we agree we have missed the mark with our initial tone. We have made edits throughout the abstract and introduction to better promote a lens of support and screening, rather than identification. We were attempting to emphasize the high prevalence of concerns and need for novel approaches in universal screening.

3. Can you provide more description of the clinician evaluation?

Response: The evaluation was a standard intake assessment as per the counsellor’s typical practice. There were two clinicians who regularly perform intake assessments as per their job. They had a conversation with each participant and this process was kept as close to real world conditions, so long as they could answer the equivalent HMU questions. They then took their evaluations and assessed participants based on their perceived degree of support needed in each domain of HEARTSMAP-U. A description of this has been added to the first paragraph of the methods section on page 5.

4. Page 10. When you discuss “sensitivity,” you mean in comparison with the dichotomous conclusions of the clinician assessment? It would be helpful to restate that here. Those clinician assessments are also open to debate, particularly the dichotomization. It’s important to address that in the Methods, prior to the statistics section, providing a justification for the reference standard.

Response: The sensitivity in this case is the tool’s psychometric performance. The ability to detect issues which require follow up as compared to an intake assessment. I have added a sentence to clarify this just prior to Table 2 in Results on page 10

5. I can’t tell what rating was considered a “concern” for comparison to the reference standard.

Response: A “concern” was any degree of unmet need (ie: not zero concern) in a psychosocial domain, which required follow up but no appropriate follow up plan was currently in place. For example, someone could be living with GAD, but felt well supported by their psychiatrist, counsellor, or community, in which case there would be “no concern”. Universal screening is not necessarily about fixing, but about detection and prevention. Each of the mild, moderate, and severe degrees of need fell under the heading of “concern”, which were rated by both the tool as well as the clinician during intake. Please refer to the second paragraph of the “Measures” section for further description of this.

6. The concept of “finding a problem and fixing it” may not be the best frame. It’s probably better to conceptualize this project as: 1) destigmatizing mental health; 2) promotion self-awareness of mental health; 3) emphasizing the importance of mental health; and 4) establishing a culture and infrastructure for mental health support including useful self-help tools.

Response: I hope our adjustments to some of the language in the article (particularly through the abstract and introduction) better reflect our agreement with you. We have been working towards the final step in your proposed sequence, whereby universal self-screening would become a part of the infrastructure that helps to empower the individuals to seek help when unmet need exists.

**Reviewer #2:**
1. Z-tests and ANOVA are mentioned but not clearly linked to specific comparisons.

Response: We apologize for misrepresenting the statistical analysis - this section of the methods was erroneously carried over from a previous iteration, and has been deleted accordingly. We calculated 95% confidence intervals via the Clopper-Pearson method in our R stats package, which is now clearly stated in the last paragraph of the “Statistical Analyses” section

2. The authors attribute a 12.6% reduction in clinician-identified mental health concerns partly to tool-driven help-seeking. However, without a control group, regression to the mean, spontaneous remission, or seasonal academic variation cannot be excluded. The causal language used in the Discussion overreaches the observational design.

Response: This is a fair assessment, and admittedly, this was not a part of our primary objective. Given the trends of the help seeking process we hypothesize this to be at least partly responsible. This was based on the 42% of participants at follow up stating that their baseline study prompted them to reach out to their health care team, but regardless, your point is well taken. We agree this was over-stated and was not our main study objective. We have made significant alterations to the fourth paragraph of the discussion to reflect this on page 14.

3. Performance varies substantially by domain (e.g., specificity 41% in Education vs 93% in Substance Use; Table 2), yet the discussion centers predominantly on psychiatric screening. Domain-specific implications are underdeveloped.

Response: We agree with you. The emphasis on the mental health domain was under-stated in the methods, and we have added a third paragraph in “Statistical Analysis” to reflect this. As we came to find, the low prevalence of concern in the other domains greatly affected their sensitivity and specificity. We have also described these shortcomings with a new third paragraph of our discussion, and have indicated why much of the focus is on the mental health domain throughout the article. Finally, we have stated this as a major limitation in the first paragraph of the conclusion.

4. Was the tool compared with broader psychometric validation frameworks (e.g., COSMIN standards). If not mention why in discussion or limitations.

Response: The tool was adapted from a previously validated tool (MyHEARTSMAP) over the course of numerous expert reviews and a series of 6 focus groups to refine the tool’s language, performance, and outcomes to ensure it was accurately measuring the intended content. Versions of the tool were then tested against a series of fictional vignettes until satisfactory performance was achieved. This process can be reviewed in our previous study (Virk P, Arora R, Burt H, et al. HEARTSMAP-U: Adapting a Psychosocial Self-Screening and Resource Navigation Support Tool for Use by Post-secondary Students. *Front Psychiatry*. 2022;13:812965. doi:10.3389/fpsyt.2022.812965). I have added a sentence to reference this to a greater extent within the first paragraph under Measures on page 6.

5. ICC interpretation is problematic. Test-retest reliability across 3 months in a dynamic mental health population does not strictly measure reliability but clinical change. Discuss if that was the intentional.

Response: On further review, we agree with you, and have taken the ICC interpretation out of the article entirely.

6. The manuscript treats clinician evaluation as the reference standard but does not report inter-rater reliability, structured diagnostic tools, or blinding procedures. If clinicians had access to HEARTSMAP-U outputs, incorporation bias may have inflated agreement.

Response: Prior to beginning the evaluations, there was significant training and discussion between clinicians. They practiced on standardized example cases together during this training period. They were completely blinded to the HEARTSMAP-U outputs, and performed their assessments independently from the self-assessments, after which we coupled the data based on participant IDs and analyzed them. We have added information regarding clinician training and blinding in the first paragraph of the methods.

7. Confidence intervals for some domains are extremely wide (e.g., sexual health sensitivity 18–90%), reflecting low prevalence and unstable estimates.

Response: Please see the new statement of limitations in paragraph 3 of the discussion (page 13) in addition to the first paragraph of the conclusion which mentions why low prevalence led to poor performance in these domains, and subsequently, why the evaluation of additional domains had limited utility in this population.

8. While sensitivity is strong, specificity for psychiatric concerns is low (54% baseline; 41% at follow-up), leading to substantial false positives (Table 2 & 3, p. 11–12). PPVs are correspondingly low (e.g., 40% for mental health; 5% or lower in several domains), limiting clinical efficiency in real-world deployment where over-triage may strain services.

Response: Specificity may be low given false positives, but we do not think this will strain services. This is largely because the majority of these cases are mildly positive, typically suggesting peer support or connecting with their family doctor in the coming weeks as opposed to sending people to the emergency department. We hope that this will provide greater awareness for participants on their current status and they are able to check in with their mental health team.

9. Comparisons are largely limited to MyHEARTSMAP; additional comparison with established tools (e.g., PHQ-9, GAD-7, CCAPS psychometrics) would contextualize performance

Response: We actually did collect some data on other similar screening tools for this demographic, namely the CHQ and the SASS. These will be analyzed in an upcoming paper.

10. Selection bias: Recruitment through a mental health advocacy network likely enriched the sample for higher baseline concern.

Response: This is true. If our primary objective was looking at prevalence of concern this would be a problem. We do touch on some prevalence stats, and as a result, we have added a statement of limitation on these values in the fourth paragraph of the discussion at the top of page 14. The primary outcome was the tool’s psychometric performance, and we do not believe the difference in prevalence in this case would impair the tool.

11. The USPSTF reference (2016) is somewhat dated given more recent screening debates.

Response: We have updated this reference with the updated 2022 statement. They continue to recommend screening should be coupled with adequate follow up plans, which we were referencing previously.
