# Supplementary material for: Utility of HEARTSMAP-U for psychosocial screening and mental health resource navigation in the young adult population
Source: PLoS One. 2026 Jul 13;21(7):e0353390. doi: 10.1371/journal.pone.0353390 (PMC13362112; doi:10.1371/journal.pone.0353390)
Supplement: Attachment — Submitted filename: Responses to Reviewers 2.docx [file pone.0353390.s005.docx]

Responses to Reviewer’s Comments

Manuscript Reference Number: PONE-D-25-63524R1

**Utility of HEARTSMAP-U for psychosocial screening and mental health resource navigation in the young adult population**

Dear Dr. Souparno Mitra,

We are grateful to the reviewers and for your suggested edits and comments for improving the manuscript.

We have addressed all the reviewers’ comments and modified the manuscript as detailed below.

Thank you in advance,

Shane Murphy

**Reviewer’s Comments**

**Reviewer #1:**

1. Avoid imperative or extreme words such as “essential.”

Response: I have carefully read through the manuscript and adjusted my wording accordingly. Changes to wording are as follows:

- First sentence of the abstract changed from “tools are essential to fill the gap” to “can help to address the gap”

- In the first paragraph of page 4 I have changed the wording from “universal screening ensures…” to “universal screening can ensure…”

- In the second to last paragraph on page 16, we have changed “we can safely assume” to “we assume”

- Near the top of page 17 in the last paragraph of the “Non-labelers cohort”, we have changed the word “crucial” to “imperative” in regard to data security

2. This statement from the abstract, methods would benefit from rewriting in plain, specific language: “We reported the sensitivity and specificity of respondent self-assessments against a clinician’s evaluation - incorporating the degree of perceived needs, along with correlative resource recommendations.”

Response: You’re right, this sentence is a bit difficult to understand as is. I have broken it into two separate sentences and simplified the language to make it a bit easier to digest. This is now the last 2 sentences of the methods section in the abstract.

**Reviewer #3:**
1. Regarding the study design – It’s described as a cohort study, but no clear exposure-outcome framework was defined - Can we mention what the primary outcome was?

Response: On further review, this study would be more accurately described as a prospective validation study. This included the participation of a university cohort which was followed over a 3 month period, but the intention of the study was to validate the tool rather than to evaluate for a primary outcome in the group. I have adjusted the wording in the abstract and methods sections to better reflect this.

2. What was the rationale for a 3 month follow up duration? - why not a longer follow up frame of time

Response: While it was not examined in detail in this study, this data was also collected in order to analyze the extent to which participants accessed their curtailed resources. We believed that a 3 month period gave individuals enough time to look into these resources and engage with them if desired. Additionally, since we were working with university students, we believed there would be issues in re-engaging participants for a follow up assessment if the study spanned over multiple academic years. As such, we thought it would be best to assess participants once in the fall and once in the spring in order to review resources engagement and to not lose participants to follow up due to graduation, transfer of schools, or from aging out of eligibility.

3. Recruitment of subjects through a mental health advocacy organization could introduce a sampling bias (with over-representation of individuals seeking help) please acknowledge this selection bias explicitly.

Response: We recognize that the overall prevalence of mental health concerns and the degree of participant engagement are likely skewed as a result of our recruitment method. The main goal of the study was to compare the performance of the tool against that of a counsellor, so we did not believe that this would be affected by the selection bias. This is however important to highlight, and we have acknowledged this in our discussion. This is in the final sentence of the first paragraph on page 14.

4. I didn't see a sample size justification or a power of study calculation- could you explain that please?

Response: I apologize, the determination of sample size was done prior to my involvement in the study, therefore it was not originally included in my methods section. I have discussed this at length with the previous team, and have added two paragraphs in the Methods section in the middle of page 6 to reflect the justification. It is based on an expected sensitivity of 90% as per our previous study, with a desired confidence interval of 95% and a desired precision of 5-7% (we used 6%). When accounting for a prevalence of mental health concerns of about 20%, this gave a rough total sample size of 500 as our benchmark for reasonable precision. This is calculated by using a modified version of Cochran’s formula, with emphasis on the expected number of affected individuals. See the following paper:

https://doi.org/10.1016/j.jclinepi.2005.02.009

5. In the clinician assessment - there was a notable lack of standardization- the "Typical intake evaluation" is undefined and can threaten internal validity of the study.

Response: We recognize that the standardization is slightly limited by allowing clinicians to conduct their intake assessments as per their usual methods. However, we believed that this would allow for the most authentic real-world conditions for the study, and would allow clinicians to build rapport with participants to a greater extent rather than a more scripted or standardized assessment. The two clinicians who performed the evaluations underwent extensive training together, which included numerous case vignettes and trial runs of previous small scale HEARTSMAP-U studies to provide an element of standardization. After this training period, we were comfortable that their evaluations were fair and reproducible.

6. Remote data collection can lead to a potential measurement bias- privacy variability, reduced physician patient rapport in video appts vs in person appts

Response: We acknowledge that variability in participants' privacy during remote assessments and differences in physician–patient rapport during video appointments compared with in-person encounters may have influenced participant responses and clinician ratings.

Several aspects of our study design may have reduced this risk. Both participant self-assessments and clinician evaluations were conducted remotely using standardized procedures, thereby minimizing systematic differences in data collection across participants. In addition, the primary objective of the study was to evaluate agreement between participant self-assessment and clinician assessment under real-world conditions in which remote care is increasingly common.

Nevertheless, we recognize that remote administration may affect disclosure of sensitive information and the quality of clinical interactions. These factors could influence the accuracy of both participant and clinician assessments and therefore represent a potential source of measurement bias. We have added this consideration as a final paragraph to the discussion section at the top of page 15.
