# Supplementary material for: Utility of HEARTSMAP-U for psychosocial screening and mental health resource navigation in the young adult population
Source: PLoS One. 2026 Jul 13;21(7):e0353390. doi: 10.1371/journal.pone.0353390 (PMC13362112; doi:10.1371/journal.pone.0353390)
Supplement: Peer review history

# Peer review: Decision Letter 0

**DOI:** 10.1371/journal.pone.0353390.r001

**Authors:** Souparno Mitra

17 Feb 2026

PONE-D-25-63524

Utility of HEARTSMAP-U for psychosocial screening and mental health resource navigation in the young adult population

PLOS One

Dear Dr. Murphy,

Thank you for submitting your manuscript to PLOS ONE. After careful consideration, we feel that it has merit but does not fully meet PLOS ONE’s publication criteria as it currently stands. Therefore, we invite you to submit a revised version of the manuscript that addresses the points raised during the review process.

Please submit your revised manuscript by Apr 03 2026 11:59PM. If you will need more time than this to complete your revisions, please reply to this message or contact the journal office at plosone@plos.org. When you're ready to submit your revision, log on to https://www.editorialmanager.com/pone/ and select the 'Submissions Needing Revision' folder to locate your manuscript file.

Please include the following items when submitting your revised manuscript:

- A letter that responds to each point raised by the academic editor and reviewer(s). You should upload this letter as a separate file labeled 'Response to Reviewers'.
- A marked-up copy of your manuscript that highlights changes made to the original version. You should upload this as a separate file labeled 'Revised Manuscript with Track Changes'.
- An unmarked version of your revised paper without tracked changes. You should upload this as a separate file labeled 'Manuscript'.

If you would like to make changes to your financial disclosure, please include your updated statement in your cover letter. Guidelines for resubmitting your figure files are available below the reviewer comments at the end of this letter.

If applicable, we recommend that you deposit your laboratory protocols in protocols.io to enhance the reproducibility of your results. Protocols.io assigns your protocol its own identifier (DOI) so that it can be cited independently in the future. For instructions see: https://journals.plos.org/plosone/s/submission-guidelines#loc-laboratory-protocols. Additionally, PLOS ONE offers an option for publishing peer-reviewed Lab Protocol articles, which describe protocols hosted on protocols.io. Read more information on sharing protocols at https://plos.org/protocols?utm_medium=editorial-email&utm_source=authorletters&utm_campaign=protocols.

We look forward to receiving your revised manuscript.

Kind regards,

Souparno Mitra, M.D.

Academic Editor

PLOS One

Journal Requirements:

When submitting your revision, we need you to address these additional requirements.

1.Please ensure that your manuscript meets PLOS ONE's style requirements, including those for file naming. The PLOS ONE style templates can be found at

https://journals.plos.org/plosone/s/file?id=wjVg/PLOSOne_formatting_sample_main_body.pdf and

https://journals.plos.org/plosone/s/file?id=ba62/PLOSOne_formatting_sample_title_authors_affiliations.pdf

2. We note that you have indicated that there are restrictions to data sharing for this study. PLOS only allows data to be available upon request if there are legal or ethical restrictions on sharing data publicly. For more information on unacceptable data access restrictions, please see http://journals.plos.org/plosone/s/data-availability#loc-unacceptable-data-access-restrictions.

Before we proceed with your manuscript, please address the following prompts:

a) If there are ethical or legal restrictions on sharing a de-identified data set, please explain them in detail (e.g., data contain potentially identifying or sensitive patient information, data are owned by a third-party organization, etc.) and who has imposed them (e.g., a Research Ethics Committee or Institutional Review Board, etc.). Please also provide contact information for a data access committee, ethics committee, or other institutional body to which data requests may be sent.

b) If there are no restrictions, please upload the minimal anonymized data set necessary to replicate your study findings to a stable, public repository and provide us with the relevant URLs, DOIs, or accession numbers. For a list of recommended repositories, please see

https://journals.plos.org/plosone/s/recommended-repositories. You also have the option of uploading the data as Supporting Information files, but we would recommend depositing data directly to a data repository if possible.

We will update your Data Availability statement on your behalf to reflect the information you provide.

3. Please amend either the title on the online submission form (via Edit Submission) or the title in the manuscript so that they are identical.

4. Your ethics statement should only appear in the Methods section of your manuscript. If your ethics statement is written in any section besides the Methods, please move it to the Methods section and delete it from any other section. Please ensure that your ethics statement is included in your manuscript, as the ethics statement entered into the online submission form will not be published alongside your manuscript.

5. Please remove your figures from within your manuscript file, leaving only the individual TIFF/EPS image files, uploaded separately. These will be automatically included in the reviewers’ PDF.

6. Please include your tables as part of your main manuscript and remove the individual files. Please note that supplementary tables (should remain/ be uploaded) as separate "supporting information" files.

7. If the reviewer comments include a recommendation to cite specific previously published works, please review and evaluate these publications to determine whether they are relevant and should be cited. There is no requirement to cite these works unless the editor has indicated otherwise.

Additional Editor Comments:

**Thank you for submitting your manuscript. The reviewers had some significant concerns related to your manuscript as mentioned below. Please review and resubmit for further consideration for publication.**

[Note: HTML markup is below. Please do not edit.]

Reviewers' comments:

Reviewer's Responses to Questions

**Comments to the Author**

1. Is the manuscript technically sound, and do the data support the conclusions?

The manuscript must describe a technically sound piece of scientific research with data that supports the conclusions. Experiments must have been conducted rigorously, with appropriate controls, replication, and sample sizes. The conclusions must be drawn appropriately based on the data presented.

Reviewer #1: Yes

Reviewer #2: Yes

**********

2. Has the statistical analysis been performed appropriately and rigorously?

Reviewer #1: Yes

Reviewer #2: I Don't Know

**********

3. Have the authors made all data underlying the findings in their manuscript fully available?

The PLOS Data policy requires authors to make all data underlying the findings described in their manuscript fully available without restriction, with rare exception (please refer to the Data Availability Statement in the manuscript PDF file). The data should be provided as part of the manuscript or its supporting information, or deposited to a public repository. For example, in addition to summary statistics, the data points behind means, medians and variance measures should be available. If there are restrictions on publicly sharing data—e.g. participant privacy or use of data from a third party—those must be specified.

Reviewer #1: Yes

Reviewer #2: Yes

**********

4. Is the manuscript presented in an intelligible fashion and written in standard English?

PLOS ONE does not copyedit accepted manuscripts, so the language in submitted articles must be clear, correct, and unambiguous. Any typographical or grammatical errors should be corrected at revision, so please note any specific errors here.

Reviewer #1: Yes

Reviewer #2: Yes

**********

5. Review Comments to the Author

Please use the space provided to explain your answers to the questions above. You may also include additional comments for the author, including concerns about dual publication, research ethics, or publication ethics. (Please upload your review as an attachment if it exceeds 20,000 characters)

Reviewer #1: 1. My primary concern with this study is conceptual. The study rationale and hypothesis are based on promotion of dichotomized mental health. Reading the abstract and introduction it feels like I will be reading about tactics for identifying the “mentally ill.” I put a great deal of effort into destigmatizing mental health, and a key component is to always address mental health on its continuum.

2. As I read through the tool, it looks like a self-awareness tool paired with guidance to resources. That is something altogether different from what seems to be described in the Abstract and Introduction. I suggest you move away from the framework of tools for diagnosis (dichotomization) and move towards tools for mental health promotion and support.

3. Can you provide more description of the clinician evaluation?

4. Page 10. When you discuss “sensitivity,” you mean in comparison with the dichotomous conclusions of the clinician assessment? It would be helpful to restate that here. Those clinician assessments are also open to debate, particularly the dichotomization. It’s important to address that in the Methods, prior to the statistics section, providing a justification for the reference standard.

5. I can’t tell what rating was considered a “concern” for comparison to the reference standard.

6. The concept of “finding a problem and fixing it” may not be the best frame. It’s probably better to conceptualize this project as: 1) destigmatizing mental health; 2) promotion self-awareness of mental health; 3) emphasizing the importance of mental health; and 4) establishing a culture and infrastructure for mental health support including useful self-help tools.

Reviewer #2: This prospective cohort study evaluates the diagnostic performance of HEARTSMAP-U, a digital psychosocial screening and resource navigation tool adapted for post-secondary students.

I have the following recommendations/changes:

1. Z-tests and ANOVA are mentioned but not clearly linked to specific comparisons.

2. The authors attribute a 12.6% reduction in clinician-identified mental health concerns partly to tool-driven help-seeking. However, without a control group, regression to the mean, spontaneous remission, or seasonal academic variation cannot be excluded. The causal language used in the Discussion overreaches the observational design.

3. Performance varies substantially by domain (e.g., specificity 41% in Education vs 93% in Substance Use; Table 2), yet the discussion centers predominantly on psychiatric screening. Domain-specific implications are underdeveloped.

4. Was the tool compared with broader psychometric validation frameworks (e.g., COSMIN standards). If not mention why in discussion or limitations.

5. ICC interpretation is problematic. Test-retest reliability across 3 months in a dynamic mental health population does not strictly measure reliability but clinical change. Discuss if that was the intentional.

Additional limitations to be discussed

1. The manuscript treats clinician evaluation as the reference standard but does not report inter-rater reliability, structured diagnostic tools, or blinding procedures. If clinicians had access to HEARTSMAP-U outputs, incorporation bias may have inflated agreement.

2. Confidence intervals for some domains are extremely wide (e.g., sexual health sensitivity 18–90%), reflecting low prevalence and unstable estimates.

3. While sensitivity is strong, specificity for psychiatric concerns is low (54% baseline; 41% at follow-up), leading to substantial false positives (Table 2 & 3, p. 11–12). PPVs are correspondingly low (e.g., 40% for mental health; 5% or lower in several domains), limiting clinical efficiency in real-world deployment where over-triage may strain services.

4. Comparisons are largely limited to MyHEARTSMAP; additional comparison with established tools (e.g., PHQ-9, GAD-7, CCAPS psychometrics) would contextualize performance.

5. Selection bias: Recruitment through a mental health advocacy network likely enriched the sample for higher baseline concern.

6. The USPSTF reference (2016) is somewhat dated given more recent screening debates.

**********

6. PLOS authors have the option to publish the peer review history of their article (what does this mean?). If published, this will include your full peer review and any attached files.

If you choose “no”, your identity will remain anonymous but your review may still be made public.

**Do you want your identity to be public for this peer review?**  For information about this choice, including consent withdrawal, please see our Privacy Policy.

Reviewer #1: **Yes:** David Ring

Reviewer #2: **Yes:** Nikhil Tondehal

**********

[NOTE: If reviewer comments were submitted as an attachment file, they will be attached to this email and accessible via the submission site. Please log into your account, locate the manuscript record, and check for the action link "View Attachments". If this link does not appear, there are no attachment files.]

To ensure your figures meet our technical requirements, please review our figure guidelines: https://journals.plos.org/plosone/s/figures

You may also use PLOS’s free figure tool, NAAS, to help you prepare publication quality figures: https://journals.plos.org/plosone/s/figures#loc-tools-for-figure-preparation.

NAAS will assess whether your figures meet our technical requirements by comparing each figure against our figure specifications.

# Author comment: Author response to Decision Letter 1

**DOI:** 10.1371/journal.pone.0353390.r002

28 Feb 2026

Responses to Reviewer’s Comments

Manuscript Reference Number: PONE-D-25-63524R1

Utility of HEARTSMAP-U for psychosocial screening and mental health resource navigation in the young adult population

Dear Dr. Souparno Mitra,

We are grateful to the reviewers and for your suggested edits and comments for improving the manuscript. We have made the suggested formatting changes as per your advice.

We have addressed all the reviewers’ comments and modified the manuscript as detailed below.

Thank you in advance.

Editor Comments:

1.Please ensure that your manuscript meets PLOS ONE's style requirements, including those for file naming. The PLOS ONE style templates can be found at

https://journals.plos.org/plosone/s/file?id=wjVg/PLOSOne_formatting_sample_main_body.pdf and https://journals.plos.org/plosone/s/file?id=ba62/PLOSOne_formatting_sample_title_authors_affiliations.pdf

Response: I have gone through each of these documents and meticulously corrected the formatting.

2. We note that you have indicated that there are restrictions to data sharing for this study. PLOS only allows data to be available upon request if there are legal or ethical restrictions on sharing data publicly. For more information on unacceptable data access restrictions, please see http://journals.plos.org/plosone/s/data-availability#loc-unacceptable-data-access-restrictions.

> Before we proceed with your manuscript, please address the following prompts:

> a) If there are ethical or legal restrictions on sharing a de-identified data set, please explain them in detail (e.g., data contain potentially identifying or sensitive patient information, data are owned by a third-party organization, etc.) and who has imposed them (e.g., a Research Ethics Committee or Institutional Review Board, etc.). Please also provide contact information for a data access committee, ethics committee, or other institutional body to which data requests may be sent.

> b) If there are no restrictions, please upload the minimal anonymized data set necessary to replicate your study findings to a stable, public repository and provide us with the relevant URLs, DOIs, or accession numbers. For a list of recommended repositories, please see

https://journals.plos.org/plosone/s/recommended-repositories. You also have the option of uploading the data as Supporting Information files, but we would recommend depositing data directly to a data repository if possible.

We will update your Data Availability statement on your behalf to reflect the information you provide.

Response: I have uploaded our data to a suggested repository and have made this available. It is referenced at the end of the manuscript and the url is included in the revised submission.

3. Please amend either the title on the online submission form (via Edit Submission) or the title in the manuscript so that they are identical.

Response: These are now identical.

4. Your ethics statement should only appear in the Methods section of your manuscript. If your ethics statement is written in any section besides the Methods, please move it to the Methods section and delete it from any other section. Please ensure that your ethics statement is included in your manuscript, as the ethics statement entered into the online submission form will not be published alongside your manuscript.

Response: The ethics statement is now included in the methods section and deleted from the end of the manuscript.

5. Please remove your figures from within your manuscript file, leaving only the individual TIFF/EPS image files, uploaded separately. These will be automatically included in the reviewers’ PDF.

Response: Figures have been removed from the manuscript file and have been uploaded separately.

6. Please include your tables as part of your main manuscript and remove the individual files. Please note that supplementary tables (should remain/ be uploaded) as separate "supporting information" files.

Response: Individual table files have been removed.

7. If the reviewer comments include a recommendation to cite specific previously published works, please review and evaluate these publications to determine whether they are relevant and should be cited. There is no requirement to cite these works unless the editor has indicated otherwise.

Response: Citations have been edited accordingly.

Reviewer’s Comments

Reviewer #1:

1. My primary concern with this study is conceptual. The study rationale and hypothesis are based on promotion of dichotomized mental health. Reading the abstract and introduction it feels like I will be reading about tactics for identifying the “mentally ill.” I put a great deal of effort into destigmatizing mental health, and a key component is to always address mental health on its continuum.

Response: We fully agree with you that we need to destigmatize mental health issues. The intention of the tool is to be a universal screening to check in rather than a dichotomous diagnostic tool. To ensure that the tool is reliable prior to launch, we have to assure there are no issues, false positives to drain the resource system, or blind spots. Our goal is to create a universal screening tool to help youth feel more comfortable with checking in on their mental wellness without needing an appointment or intake assessment. We have re-worded the purpose (last two lines) and discussion sections of the abstract to better reflect the intent of validating our screening tool. We have also made edits to the third paragraph of the introduction to put less emphasis on “identifying” and more towards supporting individuals, as we see your point regarding this dichotomizing language.

The psychometric evaluation of the tool has to evaluate what should necessitate further intervention. That is why we provide sensitivity and specificity against an intake assessment, that may or may not direct the user to further diagnostic evaluations.

2. As I read through the tool, it looks like a self-awareness tool paired with guidance to resources. That is something altogether different from what seems to be described in the Abstract and Introduction. I suggest you move away from the framework of tools for diagnosis (dichotomization) and move towards tools for mental health promotion and support.

Response: As mentioned above, we agree we have missed the mark with our initial tone. We have made edits throughout the abstract and introduction to better promote a lens of support and screening, rather than identification. We were attempting to emphasize the high prevalence of concerns and need for novel approaches in universal screening.

3. Can you provide more description of the clinician evaluation?

Response: The evaluation was a standard intake assessment as per the counsellor’s typical practice. There were two clinicians who regularly perform intake assessments as per their job. They had a conversation with each participant and this process was kept as close to real world conditions, so long as they could answer the equivalent HMU questions. They then took their evaluations and assessed participants based on their perceived degree of support needed in each domain of HEARTSMAP-U. A description of this has been added to the first paragraph of the methods section on page 5.

4. Page 10. When you discuss “sensitivity,” you mean in comparison with the dichotomous conclusions of the clinician assessment? It would be helpful to restate that here. Those clinician assessments are also open to debate, particularly the dichotomization. It’s important to address that in the Methods, prior to the statistics section, providing a justification for the reference standard.

Response: The sensitivity in this case is the tool’s psychometric performance. The ability to detect issues which require follow up as compared to an intake assessment. I have added a sentence to clarify this just prior to Table 2 in Results on page 10

5. I can’t tell what rating was considered a “concern” for comparison to the reference standard.

Response: A “concern” was any degree of unmet need (ie: not zero concern) in a psychosocial domain, which required follow up but no appropriate follow up plan was currently in place. For example, someone could be living with GAD, but felt well supported by their psychiatrist, counsellor, or community, in which case there would be “no concern”. Universal screening is not necessarily about fixing, but about detection and prevention. Each of the mild, moderate, and severe degrees of need fell under the heading of “concern”, which were rated by both the tool as well as the clinician during intake. Please refer to the second paragraph of the “Measures” section for further description of this.

6. The concept of “finding a problem and fixing it” may not be the best frame. It’s probably better to conceptualize this project as: 1) destigmatizing mental health; 2) promotion self-awareness of mental health; 3) emphasizing the importance of mental health; and 4) establishing a culture and infrastructure for mental health support including useful self-help tools.

Response: I hope our adjustments to some of the language in the article (particularly through the abstract and introduction) better reflect our agreement with you. We have been working towards the final step in your proposed sequence, whereby universal self-screening would become a part of the infrastructure that helps to empower the individuals to seek help when unmet need exists.

Reviewer #2:

1. Z-tests and ANOVA are mentioned but not clearly linked to specific comparisons.

Response: We apologize for misrepresenting the statistical analysis - this section of the methods was erroneously carried over from a previous iteration, and has been deleted accordingly. We calculated 95% confidence intervals via the Clopper-Pearson method in our R stats package, which is now clearly stated in the last paragraph of the “Statistical Analyses” section

2. The authors attribute a 12.6% reduction in clinician-identified mental health concerns partly to tool-driven help-seeking. However, without a control group, regression to the mean, spontaneous remission, or seasonal academic variation cannot be excluded. The causal language used in the Discussion overreaches the observational design.

Response: This is a fair assessment, and admittedly, this was not a part of our primary objective. Given the trends of the help seeking process we hypothesize this to be at least partly responsible. This was based on the 42% of participants at follow up stating that their baseline study prompted them to reach out to their health care team, but regardless, your point is well taken. We agree this was over-stated and was not our main study objective. We have made significant alterations to the fourth paragraph of the discussion to reflect this on page 14.

3. Performance varies substantially by domain (e.g., specificity 41% in Education vs 93% in Substance Use; Table 2), yet the discussion centers predominantly on psychiatric screening. Domain-specific implications are underdeveloped.

Response: We agree with you. The emphasis on the mental health domain was under-stated in the methods, and we have added a third paragraph in “Statistical Analysis” to reflect this. As we came to find, the low prevalence of concern in the other domains greatly affected their sensitivity and specificity. We have also described these shortcomings with a new third paragraph of our discussion, and have indicated why much of the focus is on the mental health domain throughout the article. Finally, we have stated this as a major limitation in the first paragraph of the conclusion.

4. Was the tool compared with broader psychometric validation frameworks (e.g., COSMIN standards). If not mention why in discussion or limitations.

Response: The tool was adapted from a previously validated tool (MyHEARTSMAP) over the course of numerous expert reviews and a series of 6 focus groups to refine the tool’s language, performance, and outcomes to ensure it was accurately measuring the intended content. Versions of the tool were then tested against a series of fictional vignettes until satisfactory performance was achieved. This process can be reviewed in our previous study (Virk P, Arora R, Burt H, et al. HEARTSMAP-U: Adapting a Psychosocial Self-Screening and Resource Navigation Support Tool for Use by Post-secondary Students. Front Psychiatry. 2022;13:812965. doi:10.3389/fpsyt.2022.812965). I have added a sentence to reference this to a greater extent within the first paragraph under Measures on page 6.

5. ICC interpretation is problematic. Test-retest reliability across 3 months in a dynamic mental health population does not strictly measure reliability but clinical change. Discuss if that was the intentional.

Response: On further review, we agree with you, and have taken the ICC interpretation out of the article entirely.

6. The manuscript treats clinician evaluation as the reference standard but does not report inter-rater reliability, structured diagnostic tools, or blinding procedures. If clinicians had access to HEARTSMAP-U outputs, incorporation bias may have inflated agreement.

Response: Prior to beginning the evaluations, there was significant training and discussion between clinicians. They practiced on standardized example cases together during this training period. They were completely blinded to the HEARTSMAP-U outputs, and performed their assessments independently from the self-assessments, after which we coupled the data based on participant IDs and analyzed them. We have added information regarding clinician training and blinding in the first paragraph of the methods.

7. Confidence intervals for some domains are extremely wide (e.g., sexual health sensitivity 18–90%), reflecting low prevalence and unstable estimates.

Response: Please see the new statement of limitations in paragraph 3 of the discussion (page 13) in addition to the first paragraph of the conclusion which mentions why low prevalence led to poor performance in these domains, and subsequently, why the evaluation of additional domains had limited utility in this population.

8. While sensitivity is strong, specificity for psychiatric concerns is low (54% baseline; 41% at follow-up), leading to substantial false positives (Table 2 & 3, p. 11–12). PPVs are correspondingly low (e.g., 40% for mental health; 5% or lower in several domains), limiting clinical efficiency in real-world deployment where over-triage may strain services.

Response: Specificity may be low given false positives, but we do not think this will strain services. This is largely because the majority of these cases are mildly positive, typically suggesting peer support or connecting with their family doctor in the coming weeks as opposed to sending people to the emergency department. We hope that this will provide greater awareness for participants on their current status and they are able to check in with their mental health team.

9. Comparisons are largely limited to MyHEARTSMAP; additional comparison with established tools (e.g., PHQ-9, GAD-7, CCAPS psychometrics) would contextualize performance

Response: We actually did collect some data on other similar screening tools for this demographic, namely the CHQ and the SASS. These will be analyzed in an upcoming paper.

10. Selection bias: Recruitment through a mental health advocacy network likely enriched the sample for higher baseline concern.

Response: This is true. If our primary objective was looking at prevalence of concern this would be a problem. We do touch on some prevalence stats, and as a result, we have added a statement of limitation on these values in the fourth paragraph of the discussion at the top of page 14. The primary outcome was the tool’s psychometric performance, and we do not believe the difference in prevalence in this case would impair the tool.

11. The USPSTF reference (2016) is somewhat dated given more recent screening debates.

Response: We have updated this reference with the updated 2022 statement. They continue to recommend screening should be coupled with adequate follow up plans, which we were referencing previously.

Attachment Submitted filename: Responses to Reviewers.docx

# Peer review: Decision Letter 1

**DOI:** 10.1371/journal.pone.0353390.r003

**Authors:** Souparno Mitra, Souparno Mitra

24 Apr 2026

PONE-D-25-63524R1

Utility of HEARTSMAP-U for psychosocial screening and mental health resource navigation in the young adult population

PLOS One

Dear Dr. Murphy,

Thank you for submitting your manuscript to PLOS ONE. After careful consideration, we feel that it has merit but does not fully meet PLOS ONE’s publication criteria as it currently stands. Therefore, we invite you to submit a revised version of the manuscript that addresses the points raised during the review process.

==============================

**ACADEMIC EDITOR:**

Please address reviewer comments and resubmit for further consideration

==============================

Please submit your revised manuscript by Jun 08 2026 11:59PM. If you will need more time than this to complete your revisions, please reply to this message or contact the journal office at plosone@plos.org. When you're ready to submit your revision, log on to https://www.editorialmanager.com/pone/ and select the 'Submissions Needing Revision' folder to locate your manuscript file.

Please include the following items when submitting your revised manuscript:

- A letter that responds to each point raised by the academic editor and reviewer(s). You should upload this letter as a separate file labeled 'Response to Reviewers'.
- A marked-up copy of your manuscript that highlights changes made to the original version. You should upload this as a separate file labeled 'Revised Manuscript with Track Changes'.
- An unmarked version of your revised paper without tracked changes. You should upload this as a separate file labeled 'Manuscript'.

If you would like to make changes to your financial disclosure, please include your updated statement in your cover letter. Guidelines for resubmitting your figure files are available below the reviewer comments at the end of this letter.

If applicable, we recommend that you deposit your laboratory protocols in protocols.io to enhance the reproducibility of your results. Protocols.io assigns your protocol its own identifier (DOI) so that it can be cited independently in the future. For instructions see: https://journals.plos.org/plosone/s/submission-guidelines#loc-laboratory-protocols. Additionally, PLOS ONE offers an option for publishing peer-reviewed Lab Protocol articles, which describe protocols hosted on protocols.io. Read more information on sharing protocols at https://plos.org/protocols?utm_medium=editorial-email&utm_source=authorletters&utm_campaign=protocols.

As the corresponding author, your ORCID iD is verified in the submission system and will appear in the published article. PLOS supports the use of ORCID, and we encourage all coauthors to register for an ORCID iD and use it as well. Please encourage your coauthors to verify their ORCID iD within the submission system before final acceptance, as unverified ORCID iDs will not appear in the published article. *Only*  the individual author can complete the verification step; PLOS staff *cannot*  verify ORCID iDs on behalf of authors.

We look forward to receiving your revised manuscript.

Kind regards,

Souparno Mitra, M.D.

Academic Editor

PLOS One

Journal Requirements:

1. If the reviewer comments include a recommendation to cite specific previously published works, please review and evaluate these publications to determine whether they are relevant and should be cited. There is no requirement to cite these works unless the editor has indicated otherwise.

2. Please review your reference list to ensure that it is complete and correct. If you have cited papers that have been retracted, please include the rationale for doing so in the manuscript text, or remove these references and replace them with relevant current references. Any changes to the reference list should be mentioned in the rebuttal letter that accompanies your revised manuscript. If you need to cite a retracted article, indicate the article’s retracted status in the References list and also include a citation and full reference for the retraction notice.

[Note: HTML markup is below. Please do not edit.]

Reviewers' comments:

Reviewer's Responses to Questions

**Comments to the Author**

1. If the authors have adequately addressed your comments raised in a previous round of review and you feel that this manuscript is now acceptable for publication, you may indicate that here to bypass the “Comments to the Author” section, enter your conflict of interest statement in the “Confidential to Editor” section, and submit your "Accept" recommendation.

Reviewer #1: All comments have been addressed

Reviewer #3: All comments have been addressed

**********

2. Is the manuscript technically sound, and do the data support the conclusions?

The manuscript must describe a technically sound piece of scientific research with data that supports the conclusions. Experiments must have been conducted rigorously, with appropriate controls, replication, and sample sizes. The conclusions must be drawn appropriately based on the data presented.

Reviewer #1: Yes

Reviewer #3: Partly

**********

3. Has the statistical analysis been performed appropriately and rigorously?

Reviewer #1: Yes

Reviewer #3: I Don't Know

**********

4. Have the authors made all data underlying the findings in their manuscript fully available?

The PLOS Data policy requires authors to make all data underlying the findings described in their manuscript fully available without restriction, with rare exception (please refer to the Data Availability Statement in the manuscript PDF file). The data should be provided as part of the manuscript or its supporting information, or deposited to a public repository. For example, in addition to summary statistics, the data points behind means, medians and variance measures should be available. If there are restrictions on publicly sharing data—e.g. participant privacy or use of data from a third party—those must be specified.

Reviewer #1: Yes

Reviewer #3: Yes

**********

5. Is the manuscript presented in an intelligible fashion and written in standard English?

PLOS ONE does not copyedit accepted manuscripts, so the language in submitted articles must be clear, correct, and unambiguous. Any typographical or grammatical errors should be corrected at revision, so please note any specific errors here.

Reviewer #1: Yes

Reviewer #3: Yes

**********

6. Review Comments to the Author

Please use the space provided to explain your answers to the questions above. You may also include additional comments for the author, including concerns about dual publication, research ethics, or publication ethics. (Please upload your review as an attachment if it exceeds 20,000 characters)

Reviewer #1: 1. Avoid imperative or extreme words such as “essential.”

2. This statement from the abstract, methods would benefit from rewriting in plain, specific language: “We reported the sensitivity and specificity of respondent self-assessments against a clinician’s evaluation - incorporating the degree of perceived needs, along with correlative resource recommendations.”

Reviewer #3: - Regarding the study design- Its described as a cohort study, but no clear exposrue-outcome framework was defined- Can we mention what the primary outcome was? What was the rationale for a 3 month follow up duration?- why not a longer follow up frame of time ?

- Recruitment of subjects through a mental health advocacy organization could introduce a sampling bias (with over-representation of individuals seeking help)- please acknowledge this selection bias explicitly.

- I didn't see a sample size justification or a power of study calculation- could you explain that please?

- In the clinician assessment- there was a notable lack of standardization- the "Typical intake evaluation" is undefined and can threaten internal validity of the study.

- Remote data collection can lead to a potential measurement bias- privacy variability, reduced physician patient rapport in video appts vs in person appts.

**********

7. PLOS authors have the option to publish the peer review history of their article (what does this mean?). If published, this will include your full peer review and any attached files.

If you choose “no”, your identity will remain anonymous but your review may still be made public.

**Do you want your identity to be public for this peer review?**  For information about this choice, including consent withdrawal, please see our Privacy Policy.

Reviewer #1: **Yes:** David Ring MD PhD

Reviewer #3: **Yes:** Arun Prasad

**********

[NOTE: If reviewer comments were submitted as an attachment file, they will be attached to this email and accessible via the submission site. Please log into your account, locate the manuscript record, and check for the action link "View Attachments". If this link does not appear, there are no attachment files.]

To ensure your figures meet our technical requirements, please review our figure guidelines: https://journals.plos.org/plosone/s/figures

You may also use PLOS’s free figure tool, NAAS, to help you prepare publication quality figures: https://journals.plos.org/plosone/s/figures#loc-tools-for-figure-preparation.

NAAS will assess whether your figures meet our technical requirements by comparing each figure against our figure specifications.

# Author comment: Author response to Decision Letter 2

**DOI:** 10.1371/journal.pone.0353390.r004

5 Jun 2026

Responses to Reviewer’s Comments

Manuscript Reference Number: PONE-D-25-63524R1

Utility of HEARTSMAP-U for psychosocial screening and mental health resource navigation in the young adult population

Dear Dr. Souparno Mitra,

We are grateful to the reviewers and for your suggested edits and comments for improving the manuscript.

We have addressed all the reviewers’ comments and modified the manuscript as detailed below.

Thank you in advance,

Shane Murphy

Reviewer’s Comments

Reviewer #1:

1. Avoid imperative or extreme words such as “essential.”

Response: I have carefully read through the manuscript and adjusted my wording accordingly. Changes to wording are as follows:

- First sentence of the abstract changed from “tools are essential to fill the gap” to “can help to address the gap”

- In the first paragraph of page 4 I have changed the wording from “universal screening ensures…” to “universal screening can ensure…”

- In the second to last paragraph on page 16, we have changed “we can safely assume” to “we assume”

- Near the top of page 17 in the last paragraph of the “Non-labelers cohort”, we have changed the word “crucial” to “imperative” in regard to data security

2. This statement from the abstract, methods would benefit from rewriting in plain, specific language: “We reported the sensitivity and specificity of respondent self-assessments against a clinician’s evaluation - incorporating the degree of perceived needs, along with correlative resource recommendations.”

Response: You’re right, this sentence is a bit difficult to understand as is. I have broken it into two separate sentences and simplified the language to make it a bit easier to digest. This is now the last 2 sentences of the methods section in the abstract.

Reviewer #3:

1. Regarding the study design – It’s described as a cohort study, but no clear exposure-outcome framework was defined - Can we mention what the primary outcome was?

Response: On further review, this study would be more accurately described as a prospective validation study. This included the participation of a university cohort which was followed over a 3 month period, but the intention of the study was to validate the tool rather than to evaluate for a primary outcome in the group. I have adjusted the wording in the abstract and methods sections to better reflect this.

2. What was the rationale for a 3 month follow up duration? - why not a longer follow up frame of time

Response: While it was not examined in detail in this study, this data was also collected in order to analyze the extent to which participants accessed their curtailed resources. We believed that a 3 month period gave individuals enough time to look into these resources and engage with them if desired. Additionally, since we were working with university students, we believed there would be issues in re-engaging participants for a follow up assessment if the study spanned over multiple academic years. As such, we thought it would be best to assess participants once in the fall and once in the spring in order to review resources engagement and to not lose participants to follow up due to graduation, transfer of schools, or from aging out of eligibility.

3. Recruitment of subjects through a mental health advocacy organization could introduce a sampling bias (with over-representation of individuals seeking help) please acknowledge this selection bias explicitly.

Response: We recognize that the overall prevalence of mental health concerns and the degree of participant engagement are likely skewed as a result of our recruitment method. The main goal of the study was to compare the performance of the tool against that of a counsellor, so we did not believe that this would be affected by the selection bias. This is however important to highlight, and we have acknowledged this in our discussion. This is in the final sentence of the first paragraph on page 14.

4. I didn't see a sample size justification or a power of study calculation- could you explain that please?

Response: I apologize, the determination of sample size was done prior to my involvement in the study, therefore it was not originally included in my methods section. I have discussed this at length with the previous team, and have added two paragraphs in the Methods section in the middle of page 6 to reflect the justification. It is based on an expected sensitivity of 90% as per our previous study, with a desired confidence interval of 95% and a desired precision of 5-7% (we used 6%). When accounting for a prevalence of mental health concerns of about 20%, this gave a rough total sample size of 500 as our benchmark for reasonable precision. This is calculated by using a modified version of Cochran’s formula, with emphasis on the expected number of affected individuals. See the following paper:

https://doi.org/10.1016/j.jclinepi.2005.02.009

5. In the clinician assessment - there was a notable lack of standardization- the "Typical intake evaluation" is undefined and can threaten internal validity of the study.

Response: We recognize that the standardization is slightly limited by allowing clinicians to conduct their intake assessments as per their usual methods. However, we believed that this would allow for the most authentic real-world conditions for the study, and would allow clinicians to build rapport with participants to a greater extent rather than a more scripted or standardized assessment. The two clinicians who performed the evaluations underwent extensive training together, which included numerous case vignettes and trial runs of previous small scale HEARTSMAP-U studies to provide an element of standardization. After this training period, we were comfortable that their evaluations were fair and reproducible.

6. Remote data collection can lead to a potential measurement bias- privacy variability, reduced physician patient rapport in video appts vs in person appts

Response: We acknowledge that variability in participants' privacy during remote assessments and differences in physician–patient rapport during video appointments compared with in-person encounters may have influenced participant responses and clinician ratings.

Several aspects of our study design may have reduced this risk. Both participant self-assessments and clinician evaluations were conducted remotely using standardized procedures, thereby minimizing systematic differences in data collection across participants. In addition, the primary objective of the study was to evaluate agreement between participant self-assessment and clinician assessment under real-world conditions in which remote care is increasingly common.

Nevertheless, we recognize that remote administration may affect disclosure of sensitive information and the quality of clinical interactions. These factors could influence the accuracy of both participant and clinician assessments and therefore represent a potential source of measurement bias. We have added this consideration as a final paragraph to the discussion section at the top of page 15.

Attachment Submitted filename: Responses to Reviewers 2.docx

# Peer review: Decision Letter 2

**DOI:** 10.1371/journal.pone.0353390.r005

**Authors:** Souparno Mitra, Souparno Mitra, Souparno Mitra

23 Jun 2026

Utility of HEARTSMAP-U for psychosocial screening and mental health resource navigation in the young adult population

PONE-D-25-63524R2

Dear Dr. Murphy,

We’re pleased to inform you that your manuscript has been judged scientifically suitable for publication and will be formally accepted for publication once it meets all outstanding technical requirements.

Within one week, you’ll receive an e-mail detailing the required amendments. When these have been addressed, you’ll receive a formal acceptance letter and your manuscript will be scheduled for publication.

An invoice will be generated when your article is formally accepted. Please note, if your institution has a publishing partnership with PLOS and your article meets the relevant criteria, all or part of your publication costs will be covered. Please make sure your user information is up-to-date by logging into Editorial Manager at Editorial Manager® and clicking the ‘Update My Information' link at the top of the page. For questions related to billing, please contact billing support.

If your institution or institutions have a press office, please notify them about your upcoming paper to help maximize its impact. If they’ll be preparing press materials, please inform our press team as soon as possible -- no later than 48 hours after receiving the formal acceptance. Your manuscript will remain under strict press embargo until 2 pm Eastern Time on the date of publication. For more information, please contact onepress@plos.org.

Kind regards,

Souparno Mitra, M.D.

Academic Editor

PLOS One

Additional Editor Comments (optional):

Reviewers' comments:

Reviewer's Responses to Questions

**Comments to the Author**

1. If the authors have adequately addressed your comments raised in a previous round of review and you feel that this manuscript is now acceptable for publication, you may indicate that here to bypass the “Comments to the Author” section, enter your conflict of interest statement in the “Confidential to Editor” section, and submit your "Accept" recommendation.

Reviewer #1: All comments have been addressed

Reviewer #3: All comments have been addressed

**********

2. Is the manuscript technically sound, and do the data support the conclusions?

The manuscript must describe a technically sound piece of scientific research with data that supports the conclusions. Experiments must have been conducted rigorously, with appropriate controls, replication, and sample sizes. The conclusions must be drawn appropriately based on the data presented.

Reviewer #1: Yes

Reviewer #3: Yes

**********

3. Has the statistical analysis been performed appropriately and rigorously?

Reviewer #1: Yes

Reviewer #3: I Don't Know

**********

4. Have the authors made all data underlying the findings in their manuscript fully available?

The PLOS Data policy requires authors to make all data underlying the findings described in their manuscript fully available without restriction, with rare exception (please refer to the Data Availability Statement in the manuscript PDF file). The data should be provided as part of the manuscript or its supporting information, or deposited to a public repository. For example, in addition to summary statistics, the data points behind means, medians and variance measures should be available. If there are restrictions on publicly sharing data—e.g. participant privacy or use of data from a third party—those must be specified.

Reviewer #1: Yes

Reviewer #3: Yes

**********

5. Is the manuscript presented in an intelligible fashion and written in standard English?

PLOS ONE does not copyedit accepted manuscripts, so the language in submitted articles must be clear, correct, and unambiguous. Any typographical or grammatical errors should be corrected at revision, so please note any specific errors here.

Reviewer #1: Yes

Reviewer #3: Yes

**********

6. Review Comments to the Author

Please use the space provided to explain your answers to the questions above. You may also include additional comments for the author, including concerns about dual publication, research ethics, or publication ethics. (Please upload your review as an attachment if it exceeds 20,000 characters)

Reviewer #1: (No Response)

Reviewer #3: Thanks for making suggested edits to article "Utility of HEARTSMAP-U for psychosocial screening and mental health resource navigation in the young adult population".

**********

7. PLOS authors have the option to publish the peer review history of their article (what does this mean?). If published, this will include your full peer review and any attached files.

If you choose “no”, your identity will remain anonymous but your review may still be made public.

**Do you want your identity to be public for this peer review?**  For information about this choice, including consent withdrawal, please see our Privacy Policy.

Reviewer #1: **Yes:** David Ring

Reviewer #3: **Yes:** Arun Prasad

**********

# Editor report: Acceptance letter

**DOI:** 10.1371/journal.pone.0353390.r006

**Authors:** Souparno Mitra, Souparno Mitra, Souparno Mitra

PONE-D-25-63524R2

PLOS One

Dear Dr. Murphy,

I'm pleased to inform you that your manuscript has been deemed suitable for publication in PLOS One. Congratulations! Your manuscript is now being handed over to our production team.

At this stage, our production department will prepare your paper for publication. This includes ensuring the following:

* All references, tables, and figures are properly cited

* All relevant supporting information is included in the manuscript submission,

* There are no issues that prevent the paper from being properly typeset

You will receive further instructions from the production team, including instructions on how to review your proof when it is ready. Please keep in mind that we are working through a large volume of accepted articles, so please give us a few days to review your paper and let you know the next and final steps.

Lastly, if your institution or institutions have a press office, please let them know about your upcoming paper now to help maximize its impact. If they'll be preparing press materials, please inform our press team within the next 48 hours. Your manuscript will remain under strict press embargo until 2 pm Eastern Time on the date of publication. For more information, please contact onepress@plos.org.

You will receive an invoice from PLOS for your publication fee after your manuscript has reached the completed accept phase. If you receive an email requesting payment before acceptance or for any other service, this may be a phishing scheme. Learn how to identify phishing emails and protect your accounts at https://explore.plos.org/phishing.

If we can help with anything else, please email us at customercare@plos.org.

Thank you for submitting your work to PLOS ONE and supporting open access.

Kind regards,

PLOS ONE Editorial Office Staff

on behalf of

Dr. Souparno Mitra

Academic Editor

PLOS One
